# TERRA expression is regulated by the telomere-binding proteins POT-1 and POT-2 in *Caenorhabditis elegans*

**DOI:** 10.1093/nar/gkad742

**Published:** 2023-09-15

**Authors:** Caterina Manzato, Luca Larini, Claudio Oss Pegorar, Maria Rosaria Dello Stritto, Katarina Jurikova, Verena Jantsch, Emilio Cusanelli

**Affiliations:** Laboratory of Cell Biology and Molecular Genetics, Department CIBIO, University of Trento, 38123, Trento, Italy; Laboratory of Cell Biology and Molecular Genetics, Department CIBIO, University of Trento, 38123, Trento, Italy; Laboratory of Cell Biology and Molecular Genetics, Department CIBIO, University of Trento, 38123, Trento, Italy; Department of Chromosome Biology, Max Perutz Laboratories, University of Vienna, Vienna Biocenter 1030, Vienna, Austria; Laboratory of Cell Biology and Molecular Genetics, Department CIBIO, University of Trento, 38123, Trento, Italy; Department of Genetics, Faculty of Natural Sciences, Comenius University in Bratislava, Ilkovičova 6, Mlynská dolina 84215, Bratislava, Slovakia; Department of Chromosome Biology, Max Perutz Laboratories, University of Vienna, Vienna Biocenter 1030, Vienna, Austria; Laboratory of Cell Biology and Molecular Genetics, Department CIBIO, University of Trento, 38123, Trento, Italy

## Abstract

Several aspects of telomere biology are regulated by the telomeric repeat-containing RNA TERRA. While TERRA expression is conserved through evolution, species-specific mechanisms regulate its biogenesis and function. Here we report on the expression of TERRA in *Caenorhabditis elegans*. We show that *C. elegans* TERRA is regulated by the telomere-binding proteins POT-1 and POT-2 which repress TERRA in a telomere-specific manner. *C. elegans* TERRA transcripts are heterogeneous in length and form discrete nuclear foci, as detected by RNA FISH, in both postmitotic and germline cells; a fraction of TERRA foci localizes to telomeres. Interestingly, in germ cells, TERRA is expressed in all stages of meiotic prophase I, and it increases during pachytene, a stage in meiosis when homologous recombination is ongoing. We used the MS2-GFP system to study the spatiotemporal dynamics of single-telomere TERRA molecules. Single particle tracking revealed different types of motilities, suggesting complex dynamics of TERRA transcripts. Finally, we unveiled distinctive features of *C. elegans* TERRA, which is regulated by telomere shortening in a telomere-specific manner, and it is upregulated in the telomerase-deficient *trt-1; pot-2* double mutant prior to activation of the alternative lengthening mechanism ALT. Interestingly, in these worms TERRA displays distinct dynamics with a higher fraction of fast-moving particles.

## Graphical Abstract

**Graphical Abstract:**
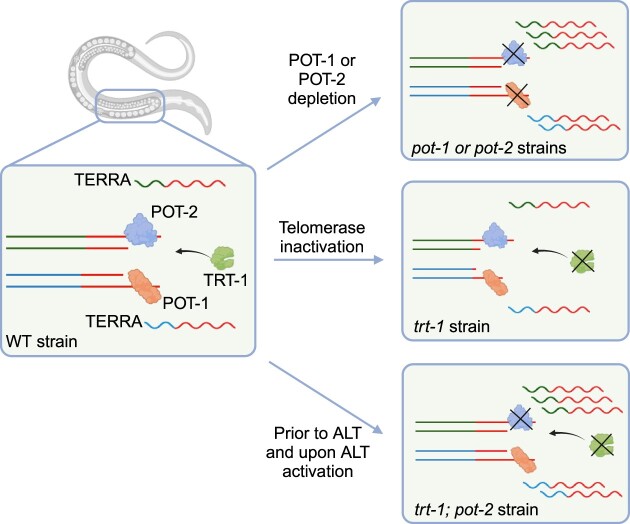

## INTRODUCTION

Telomeres are nucleoprotein structures assembled at the extremities of eukaryotic chromosomes protecting them from activation of DNA damage response pathways, degradation and erroneous recombination events (1). Telomeric DNA consists of tandem arrays of a short sequence (5′-TTAGGG-3′ in vertebrates) forming a double-stranded region terminating with a single-stranded G-rich 3′ overhang (2,3). Telomeric sequences are bound by a set of specialized proteins, forming the shelterin complex in mammals, that mediate telomere functions and determine the telomere structure (4). In humans, the shelterin complex comprises six different proteins recruited to telomeres through the direct interaction of the TRF1 and TRF2 subunits with the double-stranded telomeric sequence (5). Telomere-bound TRF1 and TRF2 proteins recruit the other shelterin components: TIN2, RAP1 and the TPP1/POT-1 heterodimer. POT-1 is a DNA binding protein specifically recognizing single-stranded telomeric DNA sequence (4).

In the absence of maintenance mechanisms, chromosome ends erode during subsequent cell divisions, a process known as the ‘end replication problem’ which is determined by the inability of the DNA replication machinery to fully replicate the ends of linear DNA molecules (6,7). Eroded telomeres become dysfunctional, and activate DNA damage response pathways, ultimately leading to cellular senescence (8). The shortening of telomeres limits the proliferative capacity of human somatic cells, acting as a barrier to tumorigenesis (9). In order to achieve full malignancy, cancer cells counteract telomere erosion by either expressing telomerase, the enzyme that reverse transcribes its RNA subunit to add telomeric DNA sequences to the extremity of chromosomes, or activating alternative telomere lengthening mechanisms, known as ALT, which rely on homologous recombination (10–13).

Despite their enrichment in heterochromatic marks, telomeres are transcribed by RNA pol II giving rise to long noncoding RNAs containing telomeric repeats termed TERRA (14,15). TERRA transcription starts from the subtelomeric region of chromosomes, terminating within the telomeric sequence (16). As so, TERRA molecules consist of subtelomeric-derived sequences at their 5′ extremity and G-rich telomeric sequences at their 3′ end. TERRA has been detected in different organisms including humans, mice, yeasts, and zebrafish (17–23). Length heterogeneity is a conserved and distinctive feature of TERRA transcripts which range from 0.1 to 9 kb in human cells (14,15,24).

The expression of TERRA is regulated by telomere-binding proteins, including TRF1 and TRF2 in humans (15,25–27), Rap1 in *Saccharomyces cerevisiae* (18,28), Rap1 and Taz1 in *Schizosaccharomyces pombe* (22,23,29), as well as *Tb*TRF in *Trypanosoma brucei* (30,31). Most TERRA molecules are nuclear, and a fraction of them localizes at telomeres (16,32). The telomeric localization of TERRA can be mediated by its interaction with telomere binding proteins, including TRF1, TRF2 and heterochromatic marks (33–36), as well as by the base pairing of TERRA transcripts with their DNA template strand, forming telomeric RNA:DNA hybrids, or R-loops (37–43). In yeast and human cells, telomeric R-loops have been shown to favor homologous recombination among telomeres (39,44). Inhibition of TERRA transcription or degradation of R-loop structures result in ALT impairment, pointing to TERRA as an important regulator of the ALT mechanism (37,45–48). In line with this evidence, TERRA expression is upregulated in ALT cancer cells, as compared to telomerase positive cells (49,50).

In addition to its role in ALT, several studies have unveiled critical functions of TERRA in i) telomeric DNA replication (51), ii) heterochromatin formation (34), iii) telomerase activity (52–54) and iv) DNA damage response pathways (25,26). These studies underline the importance of TERRA in telomere biology.

*Caenorhabditis elegans* telomeres consist of TTAGGC repeats spanning from 2 to 7 kilobases in length (55–58). The *C. elegans* genome encodes for four genes homologous to the mammalian *POT-1*: *pot-1, pot-2, pot-3 and mrt-1* (56,58), and two double-stranded telomeric DNA-binding proteins: TEBP-1 and TEBP-2 (59), also known as DTN-1 and DTN-2 (60). Telomere maintenance is carried out by the catalytic subunit of telomerase TRT-1, expressed from the *trt-1* gene (56). POT-1 and POT-2, also known as CeOB-2 and CeOB-1, respectively, act as repressors of telomerase activity (61,62). Notably, *C. elegans* represents the only multicellular organism which can survive the absence of active telomerase by activating ALT (63–65). *trt-1; pot-1* and *trt-1; pot-2* double mutants display improved survival rates compared to the *trt-1* single mutant, indicating that POT-1 and POT-2 may repress ALT (63,64). The molecular mechanisms involved in ALT in *C. elegans* remain to be defined.

Here, we report on the expression of TERRA in *C. elegans*. We show that POT-1 and POT-2 downregulate TERRA expression in a telomere-specific manner. *C. elegans* TERRA transcripts form discrete foci that are mainly nuclear, a subset of which localizes at telomeres. Interestingly, by using cytological approaches we detect TERRA foci in both postmitotic and germline cells. By studying the germline of wild type (N2), *pot-1* and *pot-2* mutant strains, we find that TERRA is expressed in all stages of meiotic prophase I and it is upregulated during pachytene, a stage of meiosis when homologous recombination is ongoing. Using a live imaging approach to study the spatiotemporal dynamics of TERRA molecules expressed from a single telomere, we show that TERRA transcripts exhibit different types of motilities: diffusive, confined, and stationary. Interestingly, TERRA particles can transit between diffusion states within single trajectories, underlining the complex dynamics of TERRA transcripts. Finally, we report that *C. elegans* TERRA is regulated by telomere shortening in a chromosome end-specific manner and it is upregulated in *trt-1; pot-2* double mutants in early generations. In these worms TERRA displays a higher fraction of fast-moving particles, as observed by live imaging. Notably, no further increase in TERRA levels is detected in three independent *trt-1; pot-2* ALT lines after several generations. These findings suggest that TERRA transcripts may participate in the onset of ALT.

## MATERIALS AND METHODS

### Experimental model and strains details

Worms were grown at 20°C on nematode growth media (NGM) plates seeded with OP50 *Escherichia coli*. The following strains and alleles were used: Wild type (N2 Bristol), *pot-2(tm1400), pot-1(tm1620), trt-1 (ok410), ypSi2 [Pdaz-1::pot-1::mCherry::tbb-2 3′UTR + Cbr-unc-119(+)] II, jf195 [5xMS2 loops::terra] I, qSi370 [mex-5p:: MS2 Coat Protein::linker::sfGFP::tbb-2 3′ UTR::gpd-2 intergenic sequence::H2B::mCherry::unc-54 3′ UTR], qSi369 [sygl-1p::24xMS2 loops::3xflag::sygl-1::sygl1 3′UTR]*. The *trt-1 (ok410)* strain was outcrossed periodically to maintain the line. All the analyses were performed on the homozygous mutant strain.

All the strains used in this study are listed in Supplementary Table S1. Genotyping was performed for each allele using the primers indicated in Supplementary Table S2.

### Northern blot

Total RNA extraction was performed using the following procedure: worms were collected by centrifugation in M9 buffer (22 mM KH_2_PO_4_, 42 mM Na_2_HPO_4_, 8,6 mM NaCl, 19 mM NH_4_Cl in water) from two 6 cm plates, containing about 500 worms. Worm pellets were frozen in liquid nitrogen and subsequently mechanically homogenized with a pestle. RNA was then extracted using TRIzol (Invitrogen) following the manufacturer instructions. RNA integrity was assessed by run on MOPS gel. 15 μg of the extracted RNA was subjected to DNase I (Invitrogen) treatment. RNase-treated control samples were treated with 2 mg/ml PureLink RNase A (Invitrogen). 15 ug of DNaseI-treated RNA were run on a denaturing gel [1.5% agarose, 1× MOPS buffer pH 7 (20 mM MOPS, 2 mM sodium acetate, 1mM EDTA) 0.7% w/w formaldehyde (Fisher Scientific), 1:10 000 Xpert Green (GRiSP)] for 4 h at 90 V. Blotting on Hybond®-N+ hybridization membrane (Cytiva/Amersham) was performed by capillarity using 10× SSC buffer pH 7 (150 mM (tri)sodium citrate dihydrate, 1.5 M sodium chloride). The RNA was crosslinked to the membrane with 120 mJ/cm^2^ with UVP CL-1000L Longwave Crosslinker (UVP 95022802). TERRA probe (5′-GCCTAAGCCTAAGCCTAAGCCTAAGCCTAA-3′) was terminally labeled with ^32^P-γATP or ^32^P-γCTP (Perkin-Elmer) using a forward phosphorylation reaction by T4 polynucleotide kinase (Thermo Fisher Scientific; EK0031) and the labeled probe was purified using Illustra Microspin G-50 columns (GE Healthcare; 11753309). The crosslinked membrane after blotting was pre-hybridized 1 h at 45°C in Church buffer (1% BSA, 1 mM EDTA, 0.5 M phosphate buffer, 7% SDS). 50 μCi of the labeled probe was added, and the hybridization was carried out overnight at 45°C. The membrane was washed twice with 0.1% SDS/2x SSC, four times with 0.1% SDS/0.2× SSC, exposed for 1–7 days and the signal was acquired on Amersham Typhoon 5 (Cytiva).

### Real-time quantitative PCR (RT-qPCR)

Total RNA was extracted as indicated in the northern blot procedure. 4 μg of the extracted RNA was subjected to DNase I (Invitrogen) treatment and 2 μg were reverse transcribed using the Superscript III Reverse Transcriptase enzyme (Invitrogen). Two separate RT reactions were performed, using oligo dT primer for amplification of the housekeeping genes (arp-6 and tba-1) and with a telomeric repeats primer (5′–GCCTAAGCCTAAGCCTAAGCCTAAGCCTAA–3′) for TERRA amplification. -RT controls were included in the experiments.

Quantitative real-time PCR reactions were performed using the 2x qPCRBIO SyGreen Mix Separate ROX (PCR Biosystems) on BioRad CFX96 and BioRad CFX384 machines.

qPCR primers were designed using subtelomeric sequences available from the JBrowse genome browser (https://jbrowse.org/jb2/) using Primer-BLAST (https://www.ncbi.nlm.nih.gov/tools/primer-blast/) and their binding sites and unique amplicons were manually confirmed using the BLAST tool on Wormbase (https://wormbase.org/tools/blast_blat). Primer efficiencies were tested by qPCR reactions using five serial dilutions of the *C. elegans* genomic DNA. The log(dilution) was plotted in function of the threshold cycle (CT). A linear regression line was determined and the R2 was calculated to understand how well the experimental data fit the regression line. The linear regression line slope was used to calculate the primer efficiency, as follows: *E* (%) = (10 – (1/slope) – 1) × 100.

The qPCR reactions have been performed in duplicates/triplicates, the mean and the standard deviation of triplicates/duplicates CT values have been calculated. For each sample, the difference between the mean of the sample CT and the mean of the housekeeping gene primers CT has been calculated (ΔCT), the error of the ΔCT (*e*) corresponds to the square root of the quadratic sum of the respective CT means. Finally, the 2^− ΔCT^ has been calculated and its error corresponds to *i* = *e* × 2^− ΔCT^/ΔCT.

Sequences of qPCR primers are shown in Supplementary Table S3. Primer efficiencies are indicated in Supplementary Table S4.

### RNA fluorescence *in situ* hybridization (RNA FISH)

Early adult worms (20 h post L4 stage) were cut in M9 buffer (22 mM KH_2_PO_4_, 42 mM Na_2_HPO_4_, 8.6 mM NaCl, 19 mM NH_4_Cl in water) to expose the gonads. 30 bodies for each sample were collected in one 1.5 ml tube. In the tube, samples were fixed with 4% v/v PFA in PBS-T (0.1% Tween in 1× PBS) and permeabilized overnight in 70% v/v ethanol at 4°C on a rotor. Samples were washed twice with 2× SSC-T [0.1% Tween-20 in 2× SSC (0.3 M NaCl, 30 mM (tri)sodium citrate dihydrate)]. Control RNase A treatment samples were treated with 2 mg/ml PureLink RNase A (Invitrogen) in 2× SSC and incubated for 2 h at 37°C. Samples not treated with RNase were incubated in 2× SSC for 2 h at 37°C. Samples were washed with wash buffer (10% v/v formamide, 2× SSC in nuclease free water), and then incubated with the telomeric repeat probe (5′-CTAAGCCTAAGCCTAAGCCTAA-3′), conjugated with the 6-FAM fluorophore at the 3′ and 5′ ends, at the final concentration of 2 μM, in hybridization buffer [1% w/v dextran sulphate, 10 mg/ml of *E. coli* tRNA (Roche), 2 mM vanadyl ribonucleoside complex (VRC) (New England BioLabs), 0.2 mg/ml RNase-free BSA (Melford), 10% v/v formamide (Fisher Bioreagents) in nuclease-free water], at 37°C overnight. Samples were washed twice with wash buffer, the first wash was performed at RT for 5 min, the second wash was performed at 37°C for 30 min, and then stained with 2 μg/ml DAPI. Samples were subsequently washed with 2× SSC and transferred to the slide using 2× SSC-T. Coverslips were mounted using Vectashield (Vector Laboratories) or Prolong Diamond Antifade (Invitrogen). All the experiments were performed in technical duplicates of biological replicates.

### Immunofluorescence (IF)

IF analyses were performed on early adult worms (20 h post L4 stage). Gonads were dissected on poly-L-lysine 0.1% w/v (Sigma-Aldrich) pre-coated slides in 10 mM (–)-tetramisole hydrochloride (Sigma-Aldrich) in PBS 1× and fixed with 4% v/v PFA in PBS 1×. Samples were covered with coverslips and frozen in liquid nitrogen. After freeze cracking (fast removal of the coverslip while the sample is still frozen) slides were permeabilized in cold methanol for 1 min. Three washes in PBS-T were performed before blocking the samples in 1% w/v BSA in PBS-T. After the blocking, slides were incubated overnight at 4°C in a humid chamber with the primary antibody. Three washes in PBS-T were performed before incubating samples with the secondary antibody for 2 h at room temperature in a humid chamber. Samples were incubated with 2 μg/ml DAPI for 1 min, washed for 20 min in PBS-T and then coverslips were mounted using Prolong Diamond Antifade Mountant (Invitrogen). All the experiments were performed in technical duplicates on biological replicates.

The list of the antibodies used in this study is indicated in Supplementary table S5.

### RNA FISH in combination with immunofluorescence

After the RNA FISH hybridization step and the two washes in wash buffer, instead of proceeding with the DAPI staining, samples were washed three times in 2× SSC-T. Samples were then blocked in 3% BSA (Melford) in 2× SSC-T for at least 30 min, up to 2 h and then incubated at 4°C overnight with the primary antibody diluted in 2× SSC-T. Samples were washed three times in 2× SSC-T and incubated with the secondary antibody diluted in 2× SSC-T, for 2 h at room temperature. Then samples were stained with 2 μg/ml DAPI. Coverslips were mounted with Vectashield (Vector Laboratories) or Prolong Diamond Antifade Mountant (Invitrogen). All the experiments were performed in technical duplicates of biological replicates.

### Image acquisition

Images were acquired using a Deltavision Ultra microscope (Cytiva) and a Nikon Eclipse Ti2 microscope equipped with the CREST Optics X-Light V2 Spinning Disk module and Video Confocal Super-resolution module. The Deltavision microscope is equipped with a sCMOS detector, 2040 × 2040 imaging array, 6.5 μm × 6.5 μm pixel size and 16-bit range. The UPlanSApo 100×/1.4 NA oil objective was used to acquire with a 0,2 μm Z-step. Images have been deconvolved with the Deltavision ultra software softWoRx (Applied Precision Inc.). Images on the Nikon Eclipse Ti2 microscope were acquired using a 100X/1.4 NA oil objective and a 0.2 μm Z-step. Three to five images were acquired to cover one entire gonad and the mosaics were generated through Adobe Photoshop or with the MosaicJ (66) plug-in of the software ImageJ (NIH). Maximum projections of the images were performed through ImageJ and then processed with Adobe Photoshop.

### Quantification and analysis of TERRA foci

Image post-processing and analyses were performed with ImageJ and a specific plug-in. For the analysis of the integrated density, average intensity and volume of TERRA and POT-1::mCherry foci, an ImageJ macro using the 3D suite plug-in was used (67). The DiAna plug-in (68) was used for the segmentation and the colocalization analyses of POT-1::mCherry and TERRA foci. This approach offers a shuffling tool that evaluates the significance of the colocalization events; only the events that were scored significant were taken into consideration in the analyses.

For the analyses of the 3D distribution of TERRA foci in the nucleus, the DiAna plug-in was used for the segmentation of TERRA foci and nuclei (visualized by DAPI) and the calculation of the distance between the center of TERRA foci and the edge, or the center, of the nucleus. By these analyses it was possible to approximate nuclei to a sphere of r radius. The radius was divided in 3 regions of equal areas as performed by Ferreira and colleagues (69). The localization of each TERRA focus within the three nuclear zones was estimated by calculating the distance of the TERRA foci center to the nuclear edge and the center of the nucleus.

### Genome editing using the CRISPR/cas9 system

A sequence of 115 bp (repair template) containing three repetitions of the MS2 sequence 5′-ACATGAGGATCACCCATGT-3′ was inserted within the subtelomeric sequence of chromosome I R in the region adjacent to the telomeric repeat tract. The repair template contained a 35 bp overlap with the subtelomeric region, acting as 5′ homology arm, and a 3′ homology arm consisting of a sequence spanning the last nucleotides of subtelomere 1R followed by telomeric sequences. For details of the repair template sequence please see Supplementary Figure S6A. Direct injections of *in vitro*-assembled Cas9-CRISPR RNA (crRNA) trans-activating crRNA (tracrRNA) ribonucleoprotein complexes into the gonad of *C. elegans* were performed as previously described (70). The following Cas9-CRISPR RNA (crRNA) was used: 5′-GATGTTGCGGTATTGGTCTTAGG-3′. Editing events were confirmed by PCR analysis. All the strains were verified by sequencing and outcrossed against wild type before analysis.

### Live imaging

Live imaging analyses were performed using early adult worms (20 h post L4 stage). Worms were mounted on a 2% w/v agarose pad and immobilized in 0.25 mM (-)-tetramisole hydrochloride (Sigma-Aldrich) in M9 buffer. In this condition worms remain immobilized for around 50 min (71). Imaging was performed in streaming with 200–600 ms intervals for 30 s with the Nikon Eclipse Ti2 microscope equipped with the CREST Optics X-Light V2 Spinning Disk and Video Confocal Super-resolution module, using a 100×/1.4 NA oil objective.

### Single particle tracking

Single particle tracking was performed using the ImageJ plugin TrackMate (72) on streaming images acquired with 200–600 ms intervals for 30 s with the Nikon Eclipse Ti2 microscope equipped with the CREST Optics X-Light V2 Spinning Disk and Video Confocal Super-resolution module, using a 100×/1.4 NA oil objective. Tracking was performed on ribonucleoproteins that remained on focus for at least 5 consecutive frames, and no gap between frames was allowed in the analyses. From the points coordinates defining the tracks, the mean square displacement (MSD) was calculated as follows: MSD = $\mathop \sum \limits_{i = 0}^n {[\sqrt {{{( {{x}_i - {x}_0} )}}^2 + {{( {{y}_i - {y}_0} )}}^2} - \sqrt {{{( {{x}_{i - 1} - {x}_0} )}}^2 + {{( {{y}_{i - 1} - {y}_0} )}}^2} ]}^2$

The diffusion coefficient *D* was calculated as the slope of the initial linear phase of the MSD plot for each focus.

### Telomere restriction fragment (TRF) and southern blot

Genomic DNA (gDNA) was extracted by incubating a pool of worms with 1 ml of lysis buffer (50 mM KCl, 1 mM Tris pH 8, 1.5 mM MgCl_2_, 0.045% Tween-20, 0.045% NP40) and 10 μl of proteinase K (Invitrogen #25530049) at 60°C for 1 h. Then, 200 μl of STE buffer (100 mM NaCl, 1 mM EDTA, 10 mM Tris/HCl pH 8.0) and 200 μl of NaCl 5M were added to the mix. After vortexing, gDNA was subjected to phenol/chloroform extraction and ethanol precipitation. For TRF reaction, 3 μg of gDNA were digested with the restriction enzyme HinfI (NEB #R0155S) and separated by overnight run in 1% agarose 0.5X TBE gel at 30 V. Prior to blotting, the gel was incubated in denaturation buffer (0.5 M NaOH, 1.5 M NaCl) for 30 min and subsequently in neutralization buffer (1.5 M Tris, 1.5 M NaCl, pH 7.5) for 30 min. Blotting was performed by capillarity on a Hybond®-N + hybridization membrane (Cytiva/Amersham) using 2× SSC buffer. The DNA was crosslinked to the membrane with 120 mJ/cm^2^ with UVP CL-1000L Longwave Crosslinker (UVP 95022802). The membrane was incubated in the hybridization buffer [5X SSC, 0,04% v/v SDS, 0.1% v/v Sarcosyl] for 1 h at 42°C and then in hybridization buffer containing the telomeric probe (5′–GCCTAAGCCTAAGCCTAAGCCTAAGCCTAA–3′) at 1 nM final concentration, overnight at 42°C. The probe was labelled with digoxigenin using the DIG Oligonucleotide 3′-End Labeling Kit, 2nd generation (Roche) according to the manufacturer instructions. After the hybridization, the membrane was washed twice in 2× SSC, 0.1% v/v SDS at RT, then twice with 0.2× SSC, 0.1% v/v SDS at RT and then twice with 0.2× SSC, 0.1% v/v SDS at 50°C. The membrane was subsequently rinsed in 2× SSC, incubated with blocking buffer [maleic buffer pH 7.5 (340 mM NaCl, 100 mM maleic acid, 180 mM NaOH), 1% w/v blocking reagent (Roche)] for 1 hour and then with Anti-Digoxigenin-AP Fab fragments (Merck), diluted 1:10 000 in blocking buffer, for up to 6 h. The membrane was then washed twice with 0.3% v/v Tween-20 (Sigma-Aldrich) in maleic buffer pH 7.5 at RT and equilibrated with AP buffer (100 mM Tris–HCl pH 9.5, 100 mM NaCl). The membrane was then incubated with CDP Star chemiluminescent substrate (Merck) diluted 1:100 in AP buffer and developed using a ChemiDoc XRS+ (BioRad) for the detection of the signal.

### Telomere restriction fragment (TRF) and southern blot coupled with pulsed-filed gel electrophoresis (PFGE)

TRF analyses of *trt-1; pot-2* mutant and ALT-like clones shown in Figure 6C were performed using PFGE coupled with radioactive Southern blot protocol. For this procedure, genomic DNA (gDNA) was isolated as described in the previous section on TRF-Southern blotting. For PFGE, 8 μg of gDNA were digested with the restriction enzyme HinfI (NEB #R0155S) and run for 13 h in 1% agarose 0.5X TBE gel using a CHEF DR III Electrophoresis Cell (Biorad #1703649) at 6 V/cm, with an included angle of 120°. The TBE buffer was kept at 14°C during the run through the CHEF cooling module. Prior to blotting, the gel was incubated in denaturation buffer (0.5 M NaOH, 1.5 M NaCl) for 30 min and subsequently in neutralization buffer (1.5 M Tris, 1.5 M NaCl, pH 7.5) for 30 min. Blotting was performed by capillarity on a Hybond®-N + hybridization membrane (Cytiva/Amersham) using 10X SSC buffer. The DNA was crosslinked to the membrane with 120 mJ/cm^2^ with UVP CL-1000L Longwave Crosslinker (UVP 95022802). Telomeric probe (5′-GCCTAAGCCTAAGCCTAAGCCTAAGCCTAA-3′) was terminally labeled with ^32^P-γATP or ^32^P-γCTP (Perkin-Elmer) using a forward phosphorylation reaction by T4 polynucleotide kinase (Thermo Fisher Scientific; EK0031) and the labeled probe was purified using Illustra Microspin G-50 columns (GE Healthcare; 11753309). The crosslinked membrane after blotting was pre-hybridized 1 hour at 45°C in Church buffer (1% BSA, 1 mM EDTA, 0.5 M phosphate buffer, 7% SDS). 50 μCi of the labeled probe was added, and the hybridization was carried out overnight at 45°C. The membrane was washed twice with 0.1% SDS/2x SSC, four times with 0.1% SDS/0.2× SSC, exposed for 1–7 days and the signal was acquired on Amersham Typhoon 5 (Cytiva).

### Isolation of ALT survivors

*trt-1(ok410); pot-2(tm1400); pot-1::mCherry* and *trt-1(ok410); pot-2(tm1400)* ALT strains were generated chunking a 0.5 × 0.5 cm chunk of plate as indicated by Cheng and colleagues (63). For this study, *trt-1(ok410); pot-2(tm1400)* ALT strains are being grown for 150 generations (15/07/2023).

### Statistical analyses

Details about statistical analyses for each figure are provided in the figure legends. Significance is indicated as follows: *P*-value: *: ≤0.05, **: ≤0.01, ***: ≤ 0.001, ****: ≤0.0001, ns: not significant. GraphPad Prism 8 was used for statistical analyses.

## RESULTS

### Detection of TERRA in *C*.*elegans*

**Figure 1.**
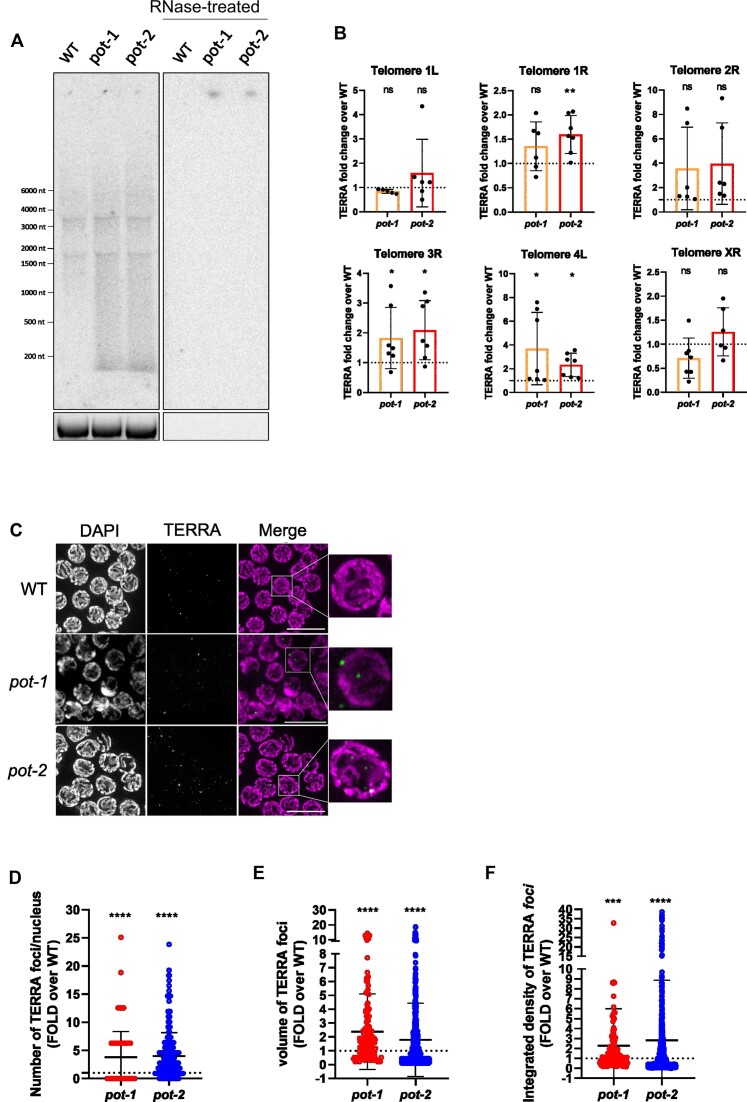
Detection of TERRA in *C. elegans*. (**A**) Northern blot analyses of TERRA from wild type (WT), *pot-1* and *pot-2* strains. Bottom image shows 18S rRNA band upon gel run. RNase A-treated samples were loaded as control for the specificity of the signal. This experiment was repeated five times obtaining similar results. (**B**) RT-qPCR analyses of TERRA expression from the indicated telomeres. Values shown represent 2^–ΔCt^ absolute values. Data is shown as fold change over the WT strain and represents mean ± SD from at least six independent biological replicates. Blank and minus reverse transcriptase controls did not produce measurable Ct values. Unpaired t-test was used to assess the statistical significance. *P*-values = *: <0.05, **: <0.01, ns: not significant. (**C**) Detection of TERRA by RNA FISH analyses in germline cells of WT, *pot-1* and *pot-2* organisms. Nuclei were stained by DAPI. A maximal projection of a z-stack experiment is shown. Scale bar: 10 μm. (**D–F**) Quantification of the TERRA foci number (D), volumes (E) and integrated density (F) per nucleus detected by RNA FISH in germline cells of WT, *pot-1* and *pot-2* organisms. Data is shown as fold over WT and represents mean ± SD from two independent experiments. Two separate analyses were performed for *pot-2* versus WT and *pot-1* versus WT. A total of four gonads for *pot-2* and its WT control were analyzed in two experiments, accounting for a total of 241 and 305 nuclei analyzed for *pot-2* and its WT, respectively; while a total of four gonads for *pot-1* and three gonads for its WT control were analyzed in two additional experiments, accounting for 368 nuclei for *pot-1* and 725 nuclei for its WT, respectively. Unpaired t-test with Welch's correction was used to assess the statistical significance. *P*-values = ***: ≤0.001, ****: ≤0.0001.

We investigated whether TERRA is expressed in *C. elegans* by performing northern blot analyses from total RNA extracted from wild type (N2) and homozygous mutant worms for the telomere binding-proteins POT-1 and POT-2 (*pot-1* and *pot-2* mutant strains). RNA hybridization using a C-rich telomeric probe enabled the detection of TERRA transcripts ranging in size from 0.1 to more than 6 kb (Figure 1A). TERRA signal was elevated in the *pot-1* and *pot-2* mutant strains, as compared to wild type. Furthermore, similar levels of TERRA were detected between the *pot-2* and *pot-1; pot-2* double mutant strain, suggesting that deficiency of both *pot-1* and *pot-2* genes does not lead to an additive effect in regulating TERRA levels (Supplementary Figure S1).

To test the expression of TERRA from distinct telomeres, we performed RT-qPCR analyses from total RNA extracted from wild type, *pot-1* and *pot-2* animals. For this approach, RNA was reverse transcribed using a C-rich telomeric primer and cDNA was analyzed by qPCR using primer pairs annealing to six different subtelomeres, in proximity of the telomeric repeat tract (Supplementary Figures S2A–C). These analyses confirmed the expression of TERRA from different telomeres. TERRA levels varied between the different strains, with a significant increase detected from two telomeres (telomeres 3R and 4L) in the *pot-1* strain and from three out of the six chromosome ends analyzed (telomeres 1R, 3R and 4L) in the *pot-2* strain (Figure 1B). These results indicate that telomere-specific mechanisms regulate TERRA expression in *pot-1* and *pot-2* strains. Furthermore, it is plausible that the transcription of multiple telomeres, including telomeres that were not analyzed by RT-qPCR, may account for the increased TERRA signal detected in *pot-1* and *pot-2* worms by northern blot, as compared to the wild type.

We used RNA fluorescence *in situ* hybridization (RNA FISH) to study TERRA expression at single cell resolution. For this approach, we employed a C-rich telomere-specific fluorescent probe to visualize TERRA transcripts expressed from all telomeres. RNA FISH analyses of wild type, *pot-1* and *pot-2* mutants revealed the formation of discrete TERRA foci (Figure 1C) which were sensitive to RNase treatment (Supplementary Figure S3A). TERRA foci were detected in the germline (Figure 1C) and in postmitotic cells of the intestine (Supplementary Figure S3B), indicating that TERRA is expressed in multiple cell types. Notably, *pot-1* and *pot-2* mutant strains displayed a higher number of TERRA foci per nucleus as compared to wild type (Figures 1D and S3C), in line with the higher TERRA levels detected in these strains. Furthermore, TERRA foci detected in the *pot-1* and *pot-2* mutants were larger and brighter than the foci detected in the wild type worms (Figure 1E and F).

Altogether, these results indicate that TERRA is expressed from multiple telomeres in *C. elegans*. TERRA expression is downregulated by POT-1 and POT-2, in a telomere-specific manner. *C. elegans* TERRA transcripts are heterogeneous in length and form discrete foci which are visualized by RNA FISH in both germline and postmitotic cells.

### TERRA expression increases in pachytene cells during meiotic prophase I

**Figure 2.**
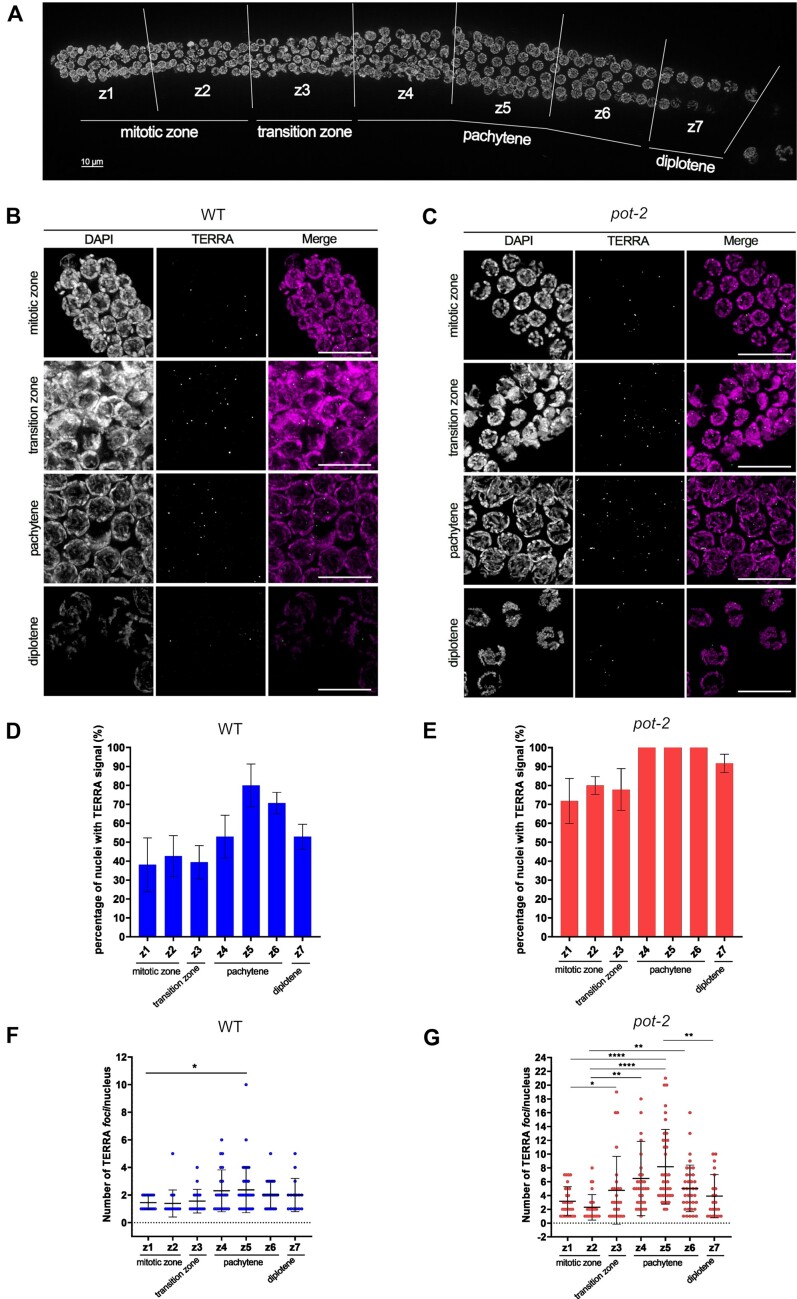
TERRA expression increases in pachytene cells during meiotic prophase I. (**A**) Low magnification image of a wild type gonad stained with DAPI depicting the seven zones of the meiotic prophase I. **(B, C)** Detection of TERRA by RNA FISH in the indicated meiotic zones of the germline of WT (B) and *pot-2* (C) organisms. Nuclei were stained by DAPI. Scale bar: 10 μm. (**D, E**) Quantification of the number of nuclei positive to TERRA signal in the different meiotic zones of WT (D) and *pot-2* (E) organisms. Data is shown as percentage of TERRA-positive nuclei and represent mean ± SD from two independent experiments. At least 240 nuclei were assessed in each experiment for each strain. Brown-Forsythe ANOVA test and the Games-Howell's multiple comparison test indicated no significant differences between zones for both strains. (**F, G**) Quantification of the number of TERRA foci per nucleus detected by RNA FISH in the indicated meiotic zones of the germline from WT (F) and *pot-2* (G) organisms. Data shown represents mean ± SD from two independent experiments. 240 nuclei were assessed in each experiment for each strain. Statistical analyses were performed using One-way Brown-Forsythe ANOVA test with column factor (difference between zones 1–7). *P*-values = **: ≤0.01 (F), *P* = ****: ≤0.0001 (G). Game-Howell's multiple comparison test (difference between single zones). *P*-values = *: ≤0.05, **: ≤0.01, ***: ≤0.001, ****: ≤0.0001

Haploid gametes are produced in the germline, where the specialized cell division program of meiosis reduces the chromosome number by half and genomes are reshuffled. During the prolonged prophase of meiosis I, programmed induction of DNA double strand breaks and their repair via homologous recombination results in the formation of a tether between the parental homologous chromosomes. The *C. elegans* hermaphrodite gonad contains mitotically dividing cells that enter meiosis and further progress through the stages of meiotic prophase I (comprising leptotene, zygotene, pachytene, diplotene and diakinesis). Cellularized oocytes in diakinesis undergo fertilization when pushed through the spermatheca, which triggers both meiotic divisions. Therefore, the gonad is organized as a distal-to-proximal assembly line that displays the stages of prophase I in a spatio-temporal order. The continuous production of progenitor cells pushes meiocytes through the gonad tube (73). To gain insight into the expression of TERRA in the germline, we performed RNA FISH analyses on dissected gonads by dividing the gonads into seven zones, roughly corresponding to the different phases of meiotic prophase I: the mitotic zone at the distal tip of the gonad, containing actively proliferating cells (z1 and z2); the transition zone, where pairing of homologous chromosomes takes place (z3); early-, mid- and late-pachytene stage (z4, z5 and z6, respectively), where crossing over occurs; the diplotene stage, where chromatin condensation results in the formation of the bivalent structures (z7) (Figure 2A) (73). Interestingly, TERRA expression was detected in all meiotic prophase I stages, in both wild type and *pot-2* mutants (Figure 2B and C). The percentage of cells positive for TERRA detection ranged from approximately 40% to 75%, in the wild type (Figure 2D), and from 70% to 100% in *pot-2* mutant worms (Figure 2E). Notably, the highest number of cells showing TERRA signal was detected during pachytene, in both the wild type and *pot-2* mutants. In the latter strain, TERRA foci were detected in all pachytene nuclei (Figure 2E).

Quantification of the number of TERRA foci per cell showed an increase from early-pachytene to mid-pachytene and a subsequent decrease during diplotene, in both wild type and *pot-2* mutants (Figure 2F and G). In the mid-pachytene of *pot-2* mutants, the number of TERRA foci per cell almost triplicated when compared to the mitotic zone. Volumes and intensities of TERRA foci remained constant throughout the different meiotic stages in wild type while they increased during pachytene in the *pot-2* strain (Supplementary Figures S4A–D). RNA FISH experiments confirmed increased detection of TERRA in pachytene cells also in the *pot-1* mutant strain (Supplementary Figures S5A–F). These results indicate that telomere transcription occurs during the various stages of the meiotic prophase I, increasing during the pachytene stage. The highest number of TERRA foci are detected during mid-pachytene when homologous recombination is ongoing.

### TERRA transcripts are mainly nuclear and localize to telomeres

**Figure 3.**
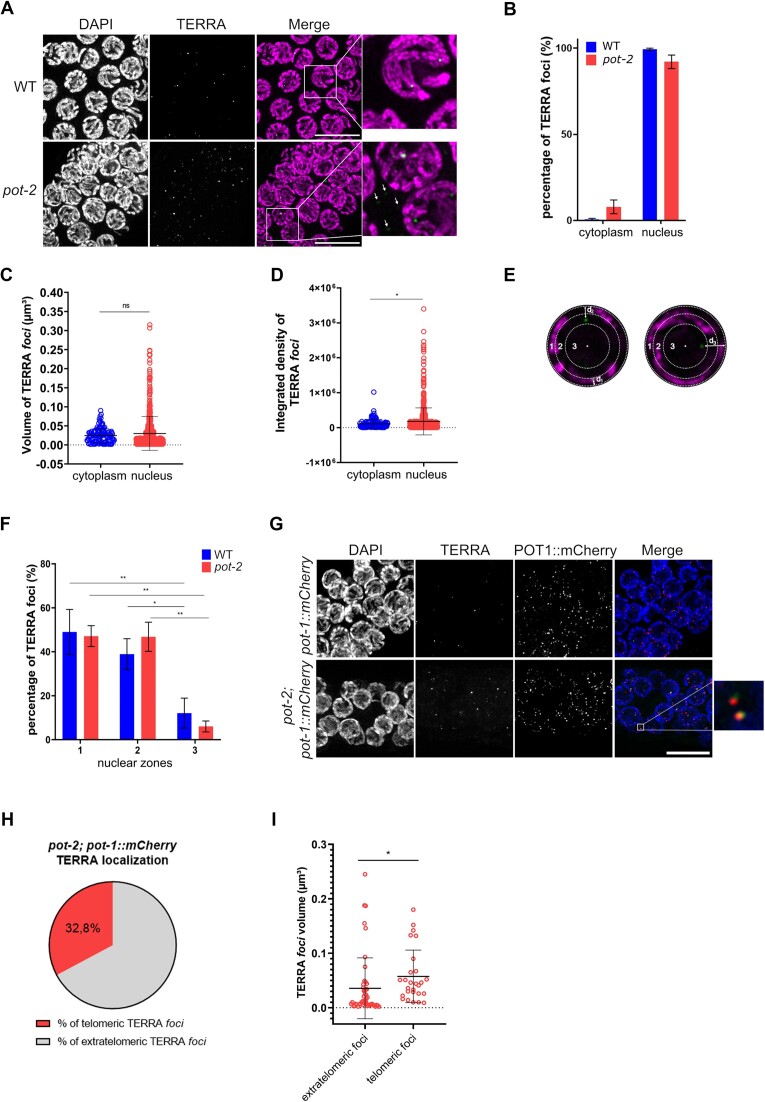
TERRA transcripts are mainly nuclear and localize at telomeres. (**A**) Detection of TERRA by RNA FISH analyses of pachytene germ cells of WT and *pot-2* organisms. Inset of the WT strain image shows representative nuclear TERRA foci. Inset of the *pot-2* strain image shows nuclear and cytoplasmic TERRA foci, the latter are indicated by white arrows. Nuclei were stained by DAPI. Scale bar: 5 μm. (**B**) Quantification of the TERRA foci detected in the nucleus or the cytoplasm in mid-pachytene cells of wild type and *pot-2* organisms. Data is shown as percentage of foci and represent mean ± SD from two independent experiments. Four gonads per genotype were analyzed, accounting for a total 131 nuclei for *pot-2* and 139 nuclei for wild type. (**C, D**) Quantification of nuclear and cytoplasmic TERRA foci volumes (C) and integrated density (D) in germline cells of pot-2 organisms. Data shown represents mean ± SD from two independent experiments, as in (B). Unpaired *t*-test with Welch's correction was used to assess the statistical significance. *P*-value = *: ≤0.05. (**E**) Representative images of nuclei showing TERRA foci localizing in the three nuclear concentric zones, as assessed by calculation of the distance of the center of each focus to the nuclear edge (d). A single plane of a 3D analysis is displayed in each image. (**F**) Quantification of the TERRA foci detected in each of the three nuclear zones in mid-pachytene germ cells of WT and *pot-2* organisms. Data is shown as percentage of foci and represent mean ± SD from two independent experiments. At least 9 nuclei were assessed for each strain in each experiment. Two-way ANOVA with Tukey's multiple comparison test was used for statistical analyses. Column factor (difference between zones 1, 2 and 3) p-value= ***: ≤0.001. Multiple comparison tests *P*-value = *: ≤0.05, **: ≤0.01. (**G**) Detection of TERRA by RNA FISH and telomeres by IF for POT1-mCherry in mid-pachytene germ cells of *pot-1::mCherry* and *pot-2*; *pot-1:mCherry* strains. Nuclei were stained by DAPI. Scale bar: 10 μm. Insets show telomeric TERRA foci defined as TERRA foci colocalizing with POT-1::mCherry foci. (**H**) Quantification of the number of telomeric or extratelomeric TERRA foci, as detected by RNA FISH/IF, shown as percentage of the total number of TERRA foci detected in mid-pachytene germ cells of *pot-2*; *pot-1::mCherry* strain. A total number of 82 TERRA foci were analyzed from 5 different gonads in two independent experiments. (**I**) Quantification of the volumes of extratelomeric and telomeric TERRA foci in mid-pachytene germ cells of the *pot-2* mutant organisms. Data represents mean ± SD from two independent experiments. At least 12 nuclei were assessed in each experiment. Unpaired t-test was used to assess the statistical significance. *P*-value = *: ≤0.05.

We used RNA FISH to gain insight into the localization of TERRA transcripts in the germline. TERRA foci were detected predominantly inside the nucleus in both the wild type and *pot-2* mutants, with a small amount of TERRA puncta localizing in the cytoplasm in *pot-2* mutants (Figure 3A and B). Nuclear TERRA foci displayed a higher intensity, although similar volumes, than cytoplasmic TERRA foci, suggesting that the latter foci consist of less or shorter TERRA transcripts (Figure 3C and D). To gain further insight into the nuclear localization of TERRA, we performed 3D segmentation of the RNA FISH images using the ImageJ software. The nuclear shape was obtained from segmentation of DAPI staining and approximated to a spherical shape through the calculation of the best radius. Three concentric zones were then defined using the method previously described by Meister and colleagues (74) (Figure 3E). We calculated the distances of the center of TERRA foci to the nearest nuclear edge in 3D, determining TERRA foci localization with respect to the three nuclear zones. Interestingly, these analyses showed that most TERRA transcripts localize at the nuclear periphery (zone 1) or to the adjacent region (zone 2), while a smaller fraction of TERRA was detected in the internal nuclear zone 3 (Figure 3F).

We next investigated whether TERRA localizes to telomeres, which reside at the nuclear periphery in *C. elegans* (69,75). To this aim, we employed a *pot-1::mCherry* strain in which the telomere-binding protein POT-1 fused to the mCherry fluorescent protein is expressed through a transgene (62). In this strain, telomeres can be visualized by fluorescence microscopy as approximately 12 discrete foci per pachytene nucleus (62). By crossing *pot-1::mCherry* worms with *pot-2* mutants, we generated a *pot-2; pot-1::mCherry* strain. Northern blot analyses revealed similar levels of TERRA between *pot-2* and *pot-2; pot1:mCherry strains*, confirming that the expression of the transgene does not affect TERRA regulation (Supplementary Figure S6A). We visualized telomeres by performing immunofluorescence (IF) experiments with an anti-mCherry antibody. Radial distribution analyses of telomeres relative to the nuclear edge of germline cells confirmed that most telomeres localize to the nuclear periphery, as previously described (69) (Supplementary Figure S6B); TERRA RNA FISH followed by IF detection of POT-1 enabled the visualization of both TERRA and telomeres in cells (Figure 3G). RNA FISH/IF analyses showed elevated levels of TERRA foci in *pot-2; pot-1::mCherry*, as compared to the *pot-1::mCherry* strain (Supplementary Figure S6C), as expected. Quantification of TERRA-telomere colocalizations revealed that nearly 33% of TERRA foci localize to telomeres in the germline of *pot-2*; *pot-1::mCherry* worms (Figure 3H). Telomeric TERRA foci were larger and brighter than the extratelomeric ones, suggesting that clustering of TERRA molecules may occur at chromosome ends (Figure 3I). No correlation was observed between the intensity and volumes of TERRA foci and the co-localizing POT-1::mCherry signal (Supplementary Figures S6D and E). Altogether, these findings indicate that *C. elegans* TERRA is predominantly nuclear and it mainly localizes close to the nuclear periphery in both wild type and *pot-2* mutants. A fraction of TERRA transcripts localizes to telomeres in the *pot-2* mutant strain, suggesting that *C. elegans* TERRA displays complex dynamic behavior in the nucleus.

### Visualization of the dynamics of single-telomere TERRA molecules in living organisms

**Figure 4.**
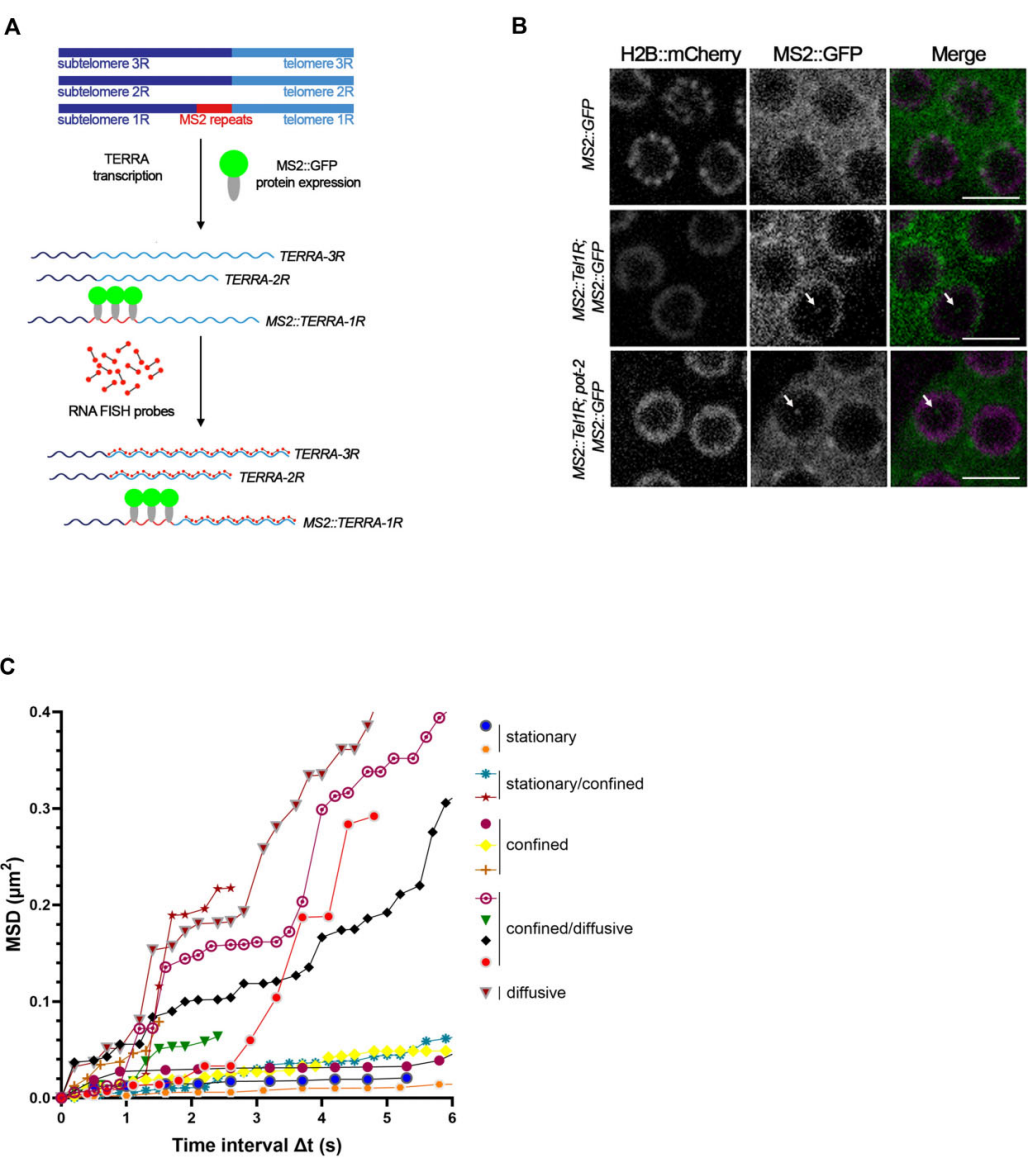
Visualization of the dynamics of single-telomere TERRA molecules in living organisms. (**A**) Schematic depiction of three *C. elegans* chromosome ends with MS2 sequences (red) integrated at subtelomere 1R, resulting in the expression of endogenous subtelomere 1R TERRA transcripts tagged with three MS2 stem loops (MS2::TERRA-1R). MS2::TERRA-1R transcripts are bound by GFP-fused MS2 coat proteins (MS2::GFP) enabling the visualization of single-telomere TERRA molecules in living cells. The use of TERRA RNA FISH probes (dumbbells with red edges) allows the visualization of TERRA molecules expressed from all telomeres in fixed cells. Only three telomeres are shown for simplicity. (**B**) Live imaging of germline cells shows TERRA-MS2-GFP foci (indicated by white arrows) in *MS2::Tel1R; MS2::GFP* and *MS2::Tel1R; pot-2; MS2::GFP* strains, but not in the *MS2::GFP* control strain. Imaging of the H2B::mCherry signal enabled the visualization of the nuclei. Scale bar: 5 μm. (**C**) Mean-square displacement (MSD) of tracked TERRA-MS2-GFP particles versus time. The tracking of 12 TERRA MS2-GFP particles in live *MS2::Tel1R; pot-2; MS2::GFP* organisms from three independent experiments is shown. Imaging was performed in streaming using a spinning disc microscope.

In order to gain information on the dynamics of TERRA transcripts in *C. elegans*, we used the MS2-GFP system to visualize endogenous TERRA molecules expressed from a single telomere in living organisms by fluorescence microscopy. This system has been previously used to tag and visualize TERRA transcripts in budding yeast and human cells (52,76–78). The MS2-GFP system relies on the high affinity binding between the MS2 RNA stem-loop sequences and the MS2 phage coat protein (79). In order to detect single-telomere TERRA transcripts in *C. elegans*, we integrated three MS2 sequences into the subtelomeric region of chromosome 1R adjacent to the telomeric repeat tract by using the CRISPR/Cas9 system (Figures 4A and S7A). With this approach, we generated the strain *MS2::Tel1R* with a homozygous integration of the MS2 sequences at subtelomere 1R, as confirmed by PCR and sequencing (Supplementary Figure S7B and C). We crossed this strain with a line expressing the MS2 RNA-binding protein fused to a super folder GFP (MS2-GFP) and an mCherry-tagged histone 2B, both driven by the *mex-5* germline promoter (80), generating the *MS2::Tel1R; MS2::GFP; H2B::mCherry* strain (hereafter *MS2::Tel1R; MS2::GFP*), homozygous for the integration of the MS2 sequences at subtelomere 1R. RT-qPCR analyses confirmed the expression of the MS2-tagged TERRA transcripts from subtelomere 1R (MS2::TERRA-1R) in the *MS2::Tel1R; MS2::GFP*, but not in the wild type or the *MS2::GFP* strain (Supplementary Figure S7D). The levels of MS2::TERRA-1R transcripts were comparable to the levels of the endogenous untagged subtelomere 1R TERRA expressed in the wild type and the MS2::GFP strain (Supplementary Figure S7E), indicating that the integration of the MS2 sequences does not influence TERRA levels.

We crossed the *MS2::Tel1R; MS2::GFP* strain with the *pot-2* mutant to generate the *MS2::Tel1R; pot-2; MS2::GFP* line homozygous for the integration of the MS2 sequences at subtelomere 1R. Live imaging revealed the formation of discrete nuclear MS2::TERRA-1R MS2::GFP (TERRA-MS2-GFP) foci, predominantly detected as a single focus per cell (Figure 4B). As expected, GFP foci were not detected in the *MS2::GFP* strain which is devoid of the MS2 sequences at the subtelomere 1R (Figure 4B). In order to confirm that the TERRA-MS2-GFP foci correspond to the TERRA foci detected by RNA FISH, we performed IF experiments using anti-GFP antibody combined with TERRA RNA FISH (Supplementary Figure S7F). Here, more than 90% of TERRA-MS2-GFP foci colocalized with the TERRA RNA FISH signal (Supplementary Figure S7G). Interestingly, TERRA-MS2-GFP foci were detected only in a fraction of nuclei displaying TERRA RNA FISH signal, with an overall 3% of TERRA RNA FISH foci colocalizing with MS2::TERRA-1R signal (Supplementary Figure S7H). These findings suggest that not all telomeres express TERRA in a cell, consistent with telomere-specific mechanisms regulating TERRA expression. Of note, we cannot exclude that TERRA molecules transcribed at low levels and not forming foci may be precluded from detection by RNA FISH/IF experiments. Together, these results confirm the specificity of the TERRA-MS2-GFP signal in *MS2::Tel1R; MS2::GFP* strains and its suitability to study TERRA dynamics.

We investigated the dynamics of TERRA-MS2-GFP foci by continuously tracking single particles by live video recording. By plotting the mean square displacement (MSD) of molecules as function of time we identified particles exhibiting distinct types of motility (Figure 4C): particles displaying an average diffusion coefficient (*D*) = 0.057 ± 0.09 μm^2^/s, indicative of diffusive motility; particles exhibiting average *D* = 0.021 ± 0.01 μm^2^/s at short times and flattening MSD curves that reached a plateau at 1–1.5 s, indicating less diffusive or confined motion; and stationary particles (average *D* = 0.005 ± 0.001 μm^2^/s) (81). Notably, as shown by their MSD curves, we observed TERRA particles transiting between different diffusion states within the measurement period of six s (Figure 4C and Supplementary Figures S8A and S8B). These findings indicate that TERRA molecules expressed from a single telomere execute complex spatiotemporal dynamics suggesting their association with different ribonucleoprotein complexes and/or localization to distinct nuclear compartments (82).

### TERRA expression is regulated by telomere shortening in a telomere-specific manner

**Figure 5.**
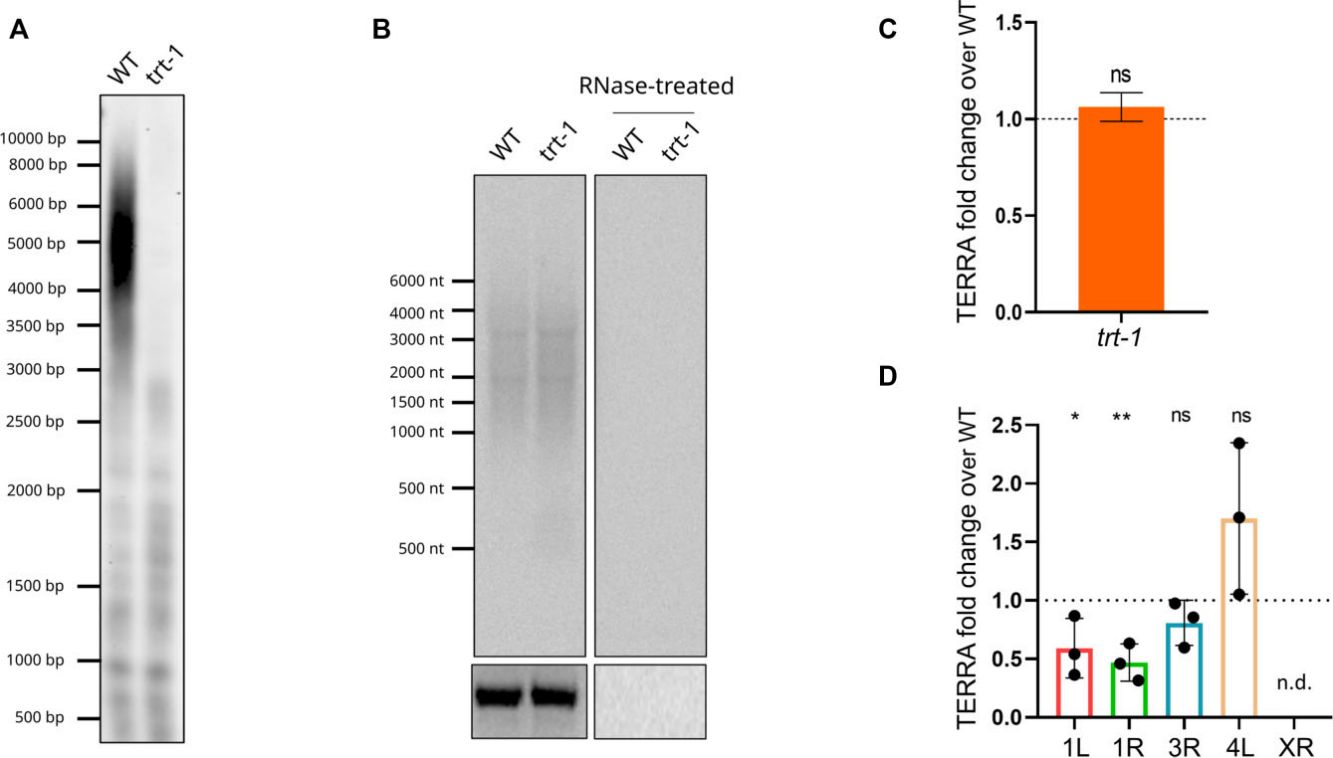
TERRA expression is regulated by telomere shortening in a telomere-specific manner. (**A**) Telomere restriction fragment analysis by Southern blotting of HinfI-digested genomic DNA extracted from WT and *trt-1* strain at F14. (**B**) Northern blot analysis of TERRA from WT and *trt-1* (F14) strains. Bottom image shows 18S rRNA band upon gel run. The experiment was repeated three independent times obtaining similar results. (**C**) Quantification of northern blots analyses of TERRA from WT and *trt-1* strains. *N* = 3. (**D**) RT-qPCR analyses of TERRA expression from the indicated telomeres in *trt-1* mutant strain (F14). Data is shown as fold change over the WT strain and represents mean and SD from three independent experiments. Two-way ANOVA with Tukey's multiple comparison test was used for statistical analyses. Column factor *P*-value = ***: ≤0.001. Multiple comparison tests *P*-value = *: ≤0.05, **: ≤0.01. n.d. = not detectable.

TERRA expression is influenced by telomere length both in humans and in yeasts (34,52,53). Indeed, telomere elongation has been shown to correlate with TERRA repression in human cells (34), while telomere shortening induces TERRA in *S. cerevisiae* and *S. pombe* (52,53). In order to gain further insight into the mechanisms regulating TERRA expression in *C. elegans*, we investigated whether telomere shortening influences TERRA levels. To this aim, we employed the telomerase-deficient *trt-1* (*ok410*) mutant which shortens telomeres during subsequent generations (56). Telomere restriction fragment (TRF) analyses confirmed the presence of eroded telomeres in *trt-1* mutant worms grown to generation 14 (F14) (Figure 5A). Northern blot analyses revealed no changes in TERRA levels in *trt-1* worms as compared to wild type (Figure 5B and C). Interestingly, RT-qPCR analyses showed no changes in TERRA expression for two out of five telomeres, while decreased TERRA levels were detected for three telomeres, with telomere XR TERRA becoming undetectable in the *trt-1* strain (Figure 5D). These findings suggest that telomere shortening does not influence the overall TERRA levels, although it is associated with telomere-specific changes in TERRA expression in *C. elegans*.

### TERRA levels and spatiotemporal dynamics are altered in *trt-1; pot-2* double mutant. ALT-like strains maintain upregulated TERRA expression compared to WT

**Figure 6.**
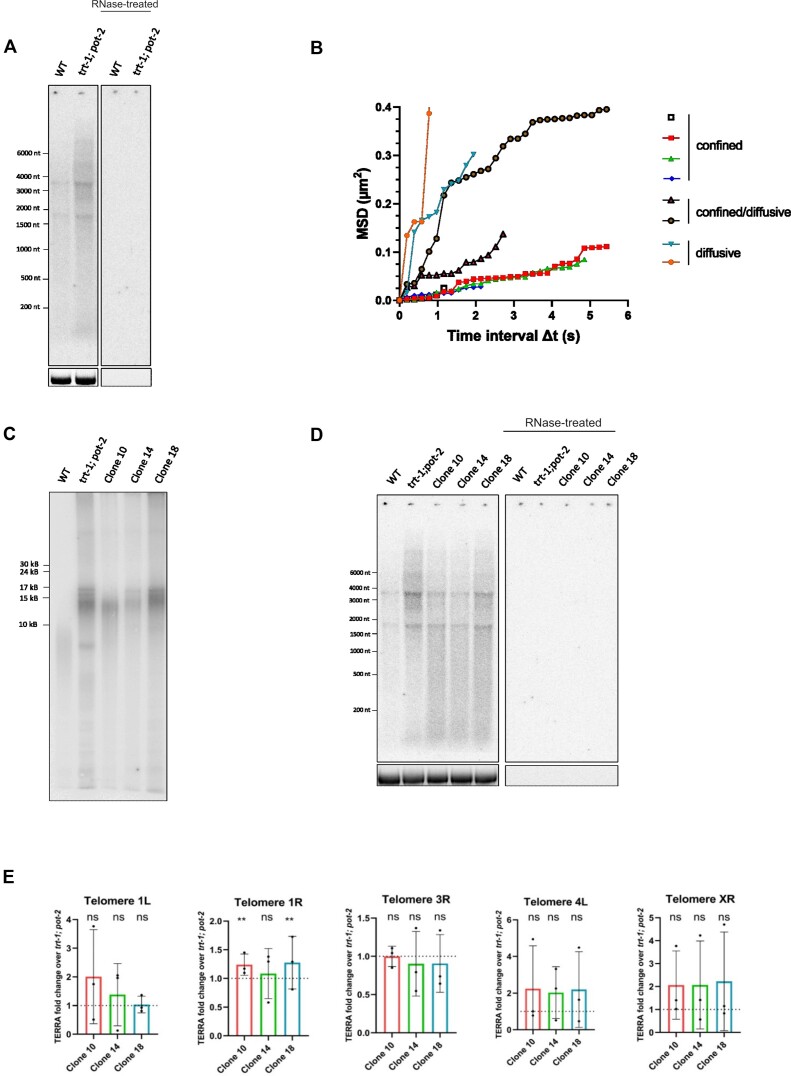
TERRA levels and spatiotemporal dynamics are altered in trt-1; pot-2 double mutant. ALT-like strains maintain upregulated TERRA expression compared to WT. (**A**) Northern blot analysis of TERRA from WT and *trt-1; pot-2* double mutant (F16). Bottom image shows 18S rRNA band upon gel run. The experiment was performed four times obtaining similar results. (**B**) Mean-square displacement (MSD) of tracked TERRA-MS2-GFP particles versus time. The tracking of 8 TERRA MS2-GFP particles in live *MS2::Tel1R; trt-1; pot-2; MS2::GFP* organisms from four independent experiments is shown. Imaging was performed in streaming using a spinning disc microscope. (**C**) Telomere restriction fragment analysis by pulsed-field gel electrophoresis (PFGE) coupled with Southern blotting of HinfI-digested genomic DNA extracted from WT, *trt-1; pot-2* (F10), and three independent *trt-1; pot-2* ALT lines, clone 10, clone 14 and clone 18 (F90). The experiment was repeated three independent times obtaining similar results. (**D**) Northern blot analysis of TERRA from WT, *trt-1; pot-2* double mutant (F16) and three independent *trt-1; pot-2* ALT lines, clone 10, clone 14 and clone 18 (F90). Bottom image shows 18S rRNA band upon gel run. The experiment was repeated three independent times obtaining similar results. (**E**) RT-qPCR analyses of TERRA from the indicated telomeres in *trt-1; pot-2* (F10), and three *trt-1; pot-2* ALT-like clones (F90). Data is shown as fold change over the *trt-1; pot-2* strain and represents mean and SD from three independent experiments. Two-way ANOVA with Tukey's multiple comparison test was used for statistical analyses. Column factor (difference between ALT-like clones) p-value = ns: not significant. Multiple comparison tests showed significant difference for telomere 1R TERRA expression between clones 10 and 18, compared to the *trt-1; pot-2* parental line. *P*-value = **: ≤0.01.

Previous evidence has shown that TERRA levels are increased in telomerase-negative human and yeast cells regulating telomere maintenance by alternative lengthening mechanisms (49,83). We thus decided to investigate TERRA expression in *C. elegans* strains maintaining telomeres by ALT mechanisms. *C. elegans* ALT strains were first developed by the Karlseder and Ahmed laboratories using *trt-1; pot-1* and *trt-1; pot-2* mutant backgrounds (63,64). To generate ALT-based survival strains, we first crossed *trt-1* with *pot-2* worms and selected homozygous *trt-1*; *pot-2* double mutant strains, in which we analyzed TERRA expression at early generations (F16). Interestingly, northern blot analyses revealed increased TERRA levels in the *trt-1; pot-2* strain as compared to the wild type (Figure 6A). On the other hand, RT-qPCR analyses showed no significant upregulation in TERRA levels from the five telomeres analyzed (Supplementary Figure S9), suggesting that the increased TERRA signal detected in *trt-1; pot-2* worms by northern blot originates from TERRA molecules transcribed from telomeres which were not analyzed by RT-qPCR. In order to gain insights into the dynamics of TERRA molecules in this genetic background, we crossed the *MS2::Tel1R; pot-2; MS2::GFP* strain with the *trt-1* mutant to generate the *MS2::Tel1R; trt-1; pot-2; MS2::GFP* line homozygous for the integration of the MS2 sequences at subtelomere 1R. As expected, live imaging revealed the formation of discrete nuclear TERRA-MS2-GFP foci, predominantly detected as a single focus per cell. Interestingly, single particle tracking of TERRA-MS2-GFP foci revealed confined and diffusive motilities, as well as particles transiting between the two different diffusion states, consistent with the results obtained in the *pot-2* mutant strain (Figure 6B). Surprisingly, no stationary particles were observed in *trt-1; pot-2* mutant organisms.

We grew the *trt-1; pot-2* strain using the ALT favorable conditions described by the Ahmed laboratory (63), consisting in transferring large agar chunks containing hundreds of worms weekly. Using these conditions, we isolated three independent *trt-1; pot-2* ‘survivors’ (clones 10, 14 and 18) which could be propagated for more than 150 generations (corresponding to 75 weeks) at 20°C. Late generation worms display a growth rate comparable to the wild type, suggesting that these lines are stably maintained. Telomere restriction fragment analysis (TRF) analyses revealed a telomere band pattern of the three clones distinct from the parental strain. Among the three clones, clone 18 showed longer telomeres, underlining differences also between clones (Figure 6C). This finding is consistent with previous evidence showing that *trt-1*; *pot-2* ALT strains can retain telomeres of different size amongst the distinct survivor isolates, which are as well distinct from the parental line (63). Notably, northern blot analyses revealed differences in TERRA levels between the three ALT lines, with clone 10 and clone 14 displaying lower levels than the *trt-1; pot-2* strain while clone 18 showing similar levels than the parental strain (Figure 6D). The three ALT lines all displayed higher levels of TERRA than the wild type (Figure 6D). When we quantified TERRA expression from five telomeres by RT-qPCR, we detected no significant changes in TERRA levels in the ALT lines, as compared to the parental strain, for most telomeres analyzed, except for telomere 1R TERRA that was found to be upregulated in clones 10 and 18 (Figure 6E). This observation is consistent with previous experiments suggesting the presence of telomere-specific regulatory mechanisms of TERRA expression in *C. elegans* (Figures 1B, 5D, Supplementary Figure S9).

Overall, these findings indicate that TERRA expression is upregulated in a *trt-1; pot-2* double mutant strain, before ALT development, in a telomere-specific manner. In *trt-1; pot-2* worms, TERRA molecules also display distinct spatiotemporal dynamics, as compared to *pot-2* mutants, with no stationary particle detected. Notably, no further increase in TERRA levels is detected in ALT strains selected when *trt-1; pot-2* are further propagated; this contrasts the observations made with yeasts and humans, where alternative lengthening of telomeres correlates with increased TERRA expression (49,83).

## DISCUSSION

In this study, we provide the first characterization of the telomeric long noncoding RNA TERRA in *C. elegans*. We show that TERRA expression is repressed by the telomere-binding proteins POT-1 and POT-2 as the total TERRA levels are elevated in *pot-1* and *pot-2* mutants compared to the wild type. Interestingly, a *pot-1; pot-2* double mutant strain expresses similar levels of TERRA as the *pot-2* single mutant, suggesting that the effect of TERRA repression of POT-1 and POT-2 is not additive, in terms of total TERRA levels. Notably, *pot-1* and *pot-2* strains retain longer telomeres than the wild type (62), which may suggest that TERRA levels correlate with telomere length in *C. elegans*. However, our findings envisage also a more complex scenario. Indeed, while the total population of TERRA is augmented in POT-1 and POT-2 depleted animals, increased TERRA expression is detected only for two out of six telomeres analyzed in the *pot-1* strain and for three out of six telomeres in the *pot-2* mutant. These results suggest that POT-1 and POT-2 regulate TERRA expression differently, depending on the transcribing telomere, in line with previous evidence indicating their distinct roles at chromosome ends (61). These findings also suggest that POT-1 and POT-2 regulate TERRA expression in a telomere-specific manner.

Notably, telomere-specific regulation of TERRA was observed also in *trt-1* mutant worms which showed no changes in the total TERRA RNA levels, as detected by northern blot, while lower TERRA levels were detected from three telomeres by RT-qPCR, compared to wild type, with telomere XR TERRA becoming undetectable in *trt-1* mutant worms. Previous evidence has shown that telomere fusions occur in the *trt-1* strain (56). The impact of these processes on TERRA expression is difficult to pinpoint at this stage. Future studies using single telomere length analyses (STELA) may provide insights into this matter. Assuming that all telomeres are similarly prone to fusions, or to complete loss of telomeric repeats due to telomere erosion, our results suggest that telomere-specific mechanisms regulate TERRA expression also at eroded telomeres in the *trt-1* mutant background. In addition, *trt-1; pot-2* double mutant resulted in increased TERRA levels detected by northern blot, as expected due to the *pot-2* deficiency. Nevertheless, no significant increase in TERRA levels from five telomeres was detected by RT-qPCR, compared to the wild type. These findings are in line with a telomere-specific regulation of TERRA in *C. elegans*.

Previous observations have shown that telomere shortening induces TERRA expression in yeasts (52,53). Furthermore, telomere-specific regulation of TERRA has been observed in humans and yeasts studies (18,84). Key determinants for this process are the presence of CpG islands and transcription factor binding sites at TERRA promoter regions of human subtelomeres (84–87) and subtelomeric repetitive DNA elements in budding yeast (28). Furthermore, recent evidence indicates that mechanisms regulating TERRA transcripts stability can significantly contribute to regulate TERRA levels in a telomere-specific manner (88,89). Future studies aimed at identifying the promoter regions of TERRA and investigating the mechanisms controlling the stability of these RNAs in *C. elegans* will help elucidate the mechanisms regulating TERRA biogenesis in this organism.

RNA FISH experiments enabled us to visualize TERRA transcripts in both germline and postmitotic cells. TERRA in postmitotic cells has been previously detected in human muscle tissues in which it may participate in the cellular response to oxidative stress during endurance exercise (90). As an interplay between TERRA expression and cellular stress has been described in different cellular and organismal contexts, from yeasts to humans (78,85,90), it will be interesting to investigate whether TERRA is influenced by cellular stress also in *C. elegans*. RNA-FISH analyses of the germline revealed that TERRA is transcribed in the mitotic zone, the sole region of the adult organism containing dividing cells, as well as in all meiotic prophase I stages. Intriguingly, in both wild type, *pot-1* and *pot-2* germlines, TERRA signal increased during pachytene, a stage of meiosis when homologous recombination is ongoing (73). Since TERRA has been shown to contribute to homologous recombination mechanisms in ALT cells, it will be interesting to investigate whether it may play a role in this mechanism during meiosis in *C. elegans* germ cells.

In this regard, by performing 3D reconstruction of pachytene nuclei, we found that TERRA is mainly localized in the intermediate and peripheric nuclear zones in wild type and *pot-2* mutant worms, indicating that a fraction of TERRA transcripts can be chromatin- and/or telomere-bound. Furthermore, these findings suggest that TERRA transcripts are probably excluded from the nucleolus which is found in the nuclear interior in pachytene cells (91). TERRA localization at the nuclear periphery has been observed also in yeast (52). Interestingly, recent findings indicate that the nuclear envelope represses telomere transcription, in both budding and fission yeasts (92,93), a process which may contribute to preventing transcription-dependent replication stress at chromosome ends (94). By employing a *pot-1*::*mCherry* strain to detect telomeres in fixed cells, we observed that 33% of TERRA foci are telomeric in the *pot-2* mutant, suggesting that TERRA is not constitutively bound to chromosome ends, at least under these experimental conditions. Indeed, we were unable to robustly assess TERRA localization to telomeres in wild type worms due to the low levels of the RNA detected in this strain. For this reason, we cannot exclude that the *pot-2* mutant background may influence TERRA localization to chromosome ends. Cytological approaches and live cell imaging have revealed that TERRA localization at telomeres is transient also in human cells and in budding yeast (50,52,76,77). In this regard, components of the nonsense mediated mRNA decay (NMD) pathway as well as heterogeneous nuclear ribonucleoproteins (hnRNPs) have been shown to participate in TERRA displacement from chromosome ends (14,95). Furthermore, TERRA associates with intrachromosomal regions to regulate gene expression in mouse embryonic stem cells (36). Thus, TERRA may exert telomeric and extratelomeric functions also in *C. elegans*. To compare the situation in nematodes, we used a live imaging approach to visualize TERRA transcripts expressed from a single telomere in living worms. Single particle tracking in *pot-2* mutant organisms revealed the presence of different populations of TERRA particles, showing distinct types of motilities. Furthermore, TERRA particles appeared to transit between different diffusion states within the same trajectories, which might suggest that they engage in interactions with different ribonucleoprotein complexes and/or localize to distinct nuclear compartment. These findings underline the complexity of the spatiotemporal dynamics of TERRA, further suggesting that telomeric transcripts may mediate multiple functions at telomeres and extratelomeric sites in *C. elegans* as well. Notably, in this study we were unable to track TERRA particles in wild type organisms. Indeed, in these worms TERRA-MS2-GFP foci displayed low intensity and signal-to-noise ratio resulting in a fast-bleaching signal that prevented particle tracking. Therefore, we cannot exclude that TERRA dynamics may be different in wild type worms.

ALT human cancer cells and telomerase-negative yeast survivors express high levels of TERRA (49,83). This condition can support telomere maintenance by homologous recombination (41,45,83). However, whether TERRA expression participates to the onset of ALT remains to be defined. *C. elegans* is an excellent model system to study this aspect of TERRA biology since ALT strains have been shown to arise spontaneously in a telomerase deficient background. Interestingly, *trt-1; pot-2*, and *trt-1; pot-1*, double mutants display elevated rates of survivors than the *trt-1* single mutant, suggesting that POT-1 and POT-2 repress ALT mechanisms (63,64). We found that *trt-1; pot-2* organisms express increased TERRA levels at early generations; however, no further increase in TERRA expression was detected in three independent ALT-based lines generated from *trt-1; pot-2* double mutants which were maintained for over 150 generations. These ALT-based lines displayed differences in the total TERRA levels although they all exhibited higher TERRA signal than wild type. Single particle tracking of TERRA molecules in a *trt-1; pot-2* double mutant genetic background revealed distinct dynamics, as compared to *pot-2*, with no stationary TERRA particles detected. The higher fraction of fast-moving TERRA particles may more frequently localize to telomeres in the *trt1; pot-2* strain. This hypothesis will need to be confirmed by further investigations. The differences in TERRA dynamics between *pot-2* and *trt-1; pot-2* mutants suggest a minor influence of the MS2 tagging approach on the RNA particles’ motility that was used for both genetic backgrounds. The upregulated TERRA levels and the dynamics of TERRA particles detected in *trt-1; pot-2* double mutants prior to ALT induction argue that TERRA may participate in the onset of ALT. In this regard, it is intriguing to speculate that TERRA may be involved in the early steps of ALT activation, for example by creating replicative stress which can promote homologous recombination at telomeres (96,97). Notably, RNA:DNA hybrids (R-loops) have been reported at telomeres in *C. elegans* embryos (98). As R-loops can perturb replication forks causing replicative stress (99), it would be important to investigate whether such structures occur at telomeres in ALT worms as well as in *pot* deficient strains. It is also notable that TERRA signal is highest during pachytene, as the meiotic recombination complex has been shown to be involved in ALT (100). Moreover, mechanisms controlling homologous recombination during meiosis play key roles in ALT (42,101–104). In future studies, the identification of TERRA promoter regions and transcripts sequences in nematodes may enable researchers to modify TERRA levels in *trt-1; pot-2* double mutant strains to test the effect on ALT induction. Similarly, the use of TERRA-targeting RNAi approaches may reveal critical for investigations of TERRA functions in ALT development (105).

The conservation of TERRA expression through evolution underlines the importance of telomere transcription, which has been confirmed by the numerous roles proposed for TERRA in several organisms. Despite the significant advances in the biology of these transcripts, the molecular mechanisms regulating their biogenesis and functions remain to be defined. The recent findings on TERRA transcripts stability regulation and post-transcriptional modifications (88,89), its role in innate immune response (106) and on its possible translation giving rise to dipeptide repeat proteins (107) highlight the importance of continuing investigations in this field. Our findings indicate that several TERRA features are conserved in *C. elegans*, these features include its length heterogeneity, complex spatiotemporal dynamics, telomeric localization, and telomere-specific regulation. Our study also brings to light distinctive features of TERRA in nematodes, where it is upregulated during pachytene and where ALT impacts its expression in a different modality as compared to humans and yeasts. Furthermore, telomere shortening does not influence the overall TERRA levels in worms. The study of TERRA in *C. elegans*, an organism amenable to cytological analyses of both proliferating and postmitotic cells, which allows the study of ALT in the context of an organism, is expected to provide important insights into the biology of TERRA and the telomere field.

## Supplementary Material

gkad742_Supplemental_file

## Data Availability

The data underlying this article will be shared on reasonable request to the corresponding author.

## Notes

Present address: Caterina Manzato, Center for Molecular Medicine, University Medical Center Utrecht 3584, The Netherlands.
