# Supplementary material for: TERRA expression is regulated by the telomere-binding proteins POT-1 and POT-2 in Caenorhabditis elegans
Source: Nucleic Acids Res. 2023 Sep 15;51(19):10681–99. doi: 10.1093/nar/gkad742 (PMC10602879; doi:10.1093/nar/gkad742)
Supplement: gkad742_Supplemental_file [file gkad742_supplemental_file.pdf]

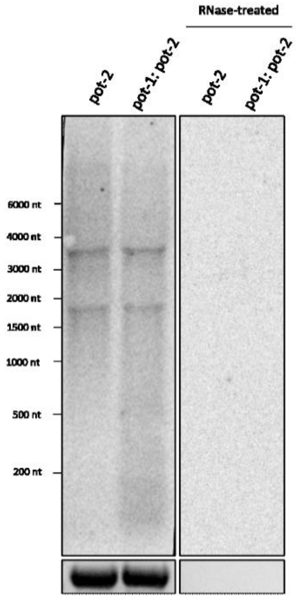

Figure S1. Quantification of TERRA expression in *pot-2* single mutant and *pot-1; pot-2* double mutant. Northern blot analysis of TERRA from *pot-2* and *pot-1; pot-2* double mutant. Total RNA was resolved on agarose gel and membrane hybridization was performed using a radioactively labelled C-rich telomeric probe. Bottom image shows 18S rRNA band upon gel run. The experiment was repeated two times obtaining similar results.

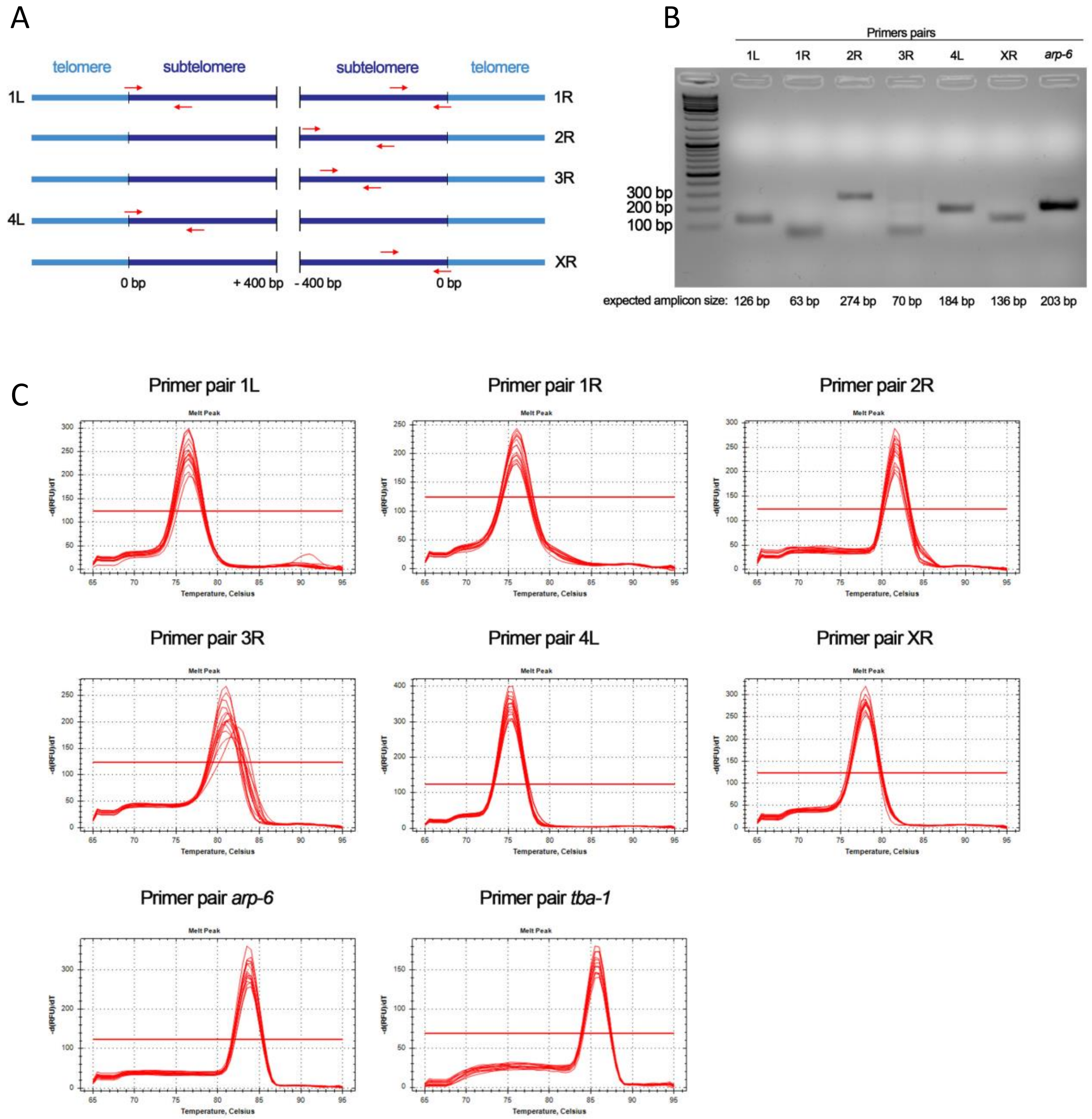

Figure S2. Validation of TERRA primers used for qPCR analyses. A) Schematic depiction of the genomic regions from which TERRA primers were designed. Subtelomeric and telomeric regions are shown in dark and light blue, respectively. TERRA primer pairs are depicted as red arrows. **B)** Amplification products from RT-qPCR analyses of RNA from wild type organisms, using TERRA qPCR primer pairs, were run on agarose gel as control of the specificity of the amplification and amplicon size. The amplification products displayed a single amplicon of the expected size for all primer pairs analyzed. The expected amplicon lengths for each primer pair are shown below the gel. Sequences of primers used in qPCR analyses are shown in Table S3. Amplification efficiencies were calculated for each primer pair and are shown in Table S4. **C)** Melting curves of primer pairs used in the RT-qPCR quantification of TERRA transcripts and reference genes (*arp-6* and *tba-1*). Additional qPCR primer pairs were tested in the attempt to detect TERRA from other telomeres, however no further primers were identified displaying reliable melting curves, adequate amplification efficiency and single amplicons (data not shown).

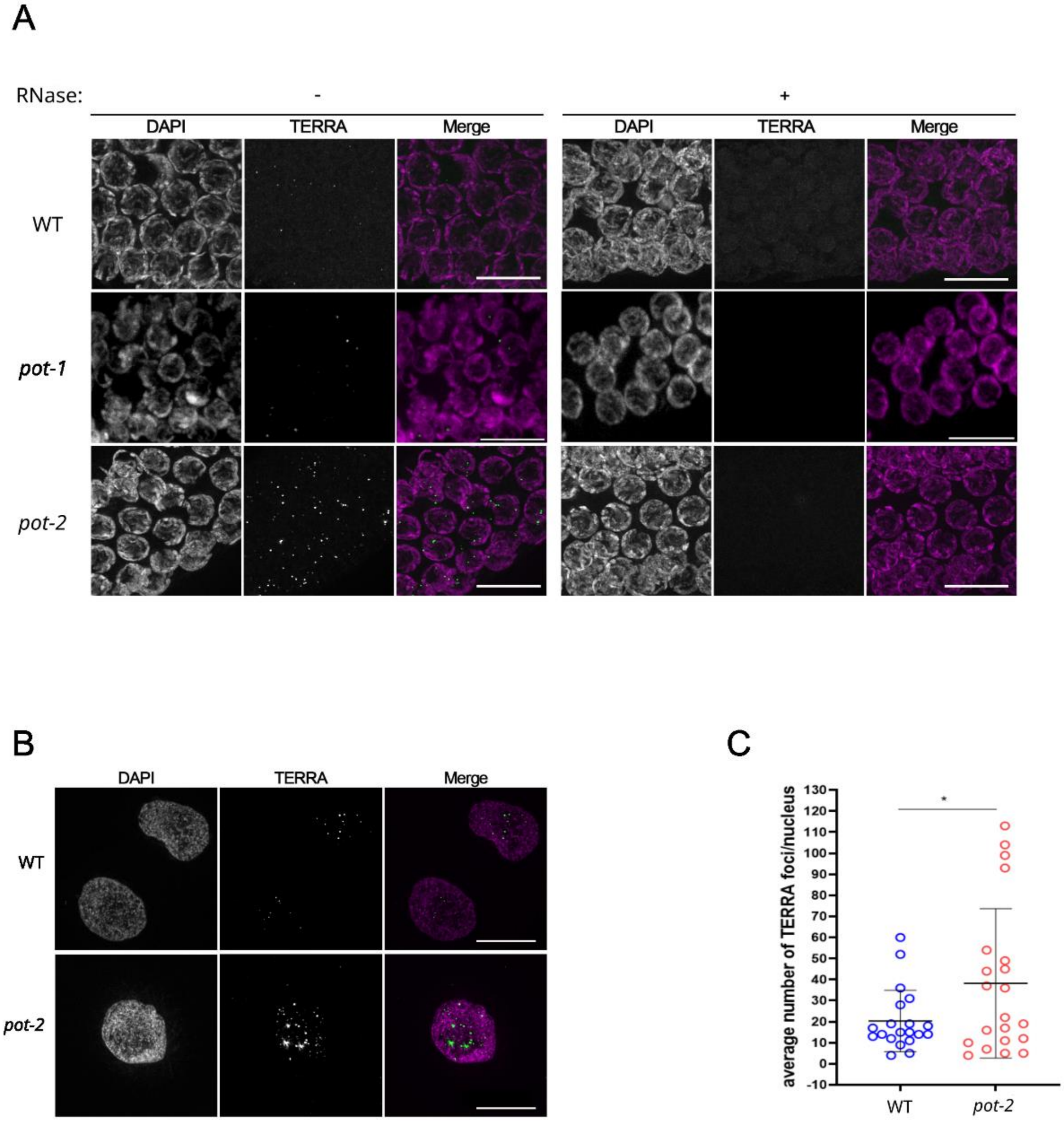

Figure S3. Validation of TERRA RNA FISH probe specificity in RNase A-treated samples and detection of TERRA in postmitotic cells. A) Detection of TERRA by RNA FISH in the germline of wild type (WT), *pot-1* and *pot-2* worms. The RNase A treatment of the samples before probe hybridization abolished TERRA signal. Nuclei were stained by DAPI. Scale bar: 10 µm. **B)** Detection of TERRA by RNA FISH in postmitotic cells of the intestine of wild type and *pot-2* organisms. Nuclei were stained by DAPI. Scale bar: 5 µm. **C)** Quantification of the number of TERRA foci per nucleus detected by RNA FISH in postmitotic cells of the intestine of wild type and *pot-2* organisms. Data shown represent mean and SD from two independent experiments. At least 20 nuclei were analyzed in each strain. Unpaired t-test with Welch's correction was used to assess the statistical significance. p-value= *: ≤0.05.

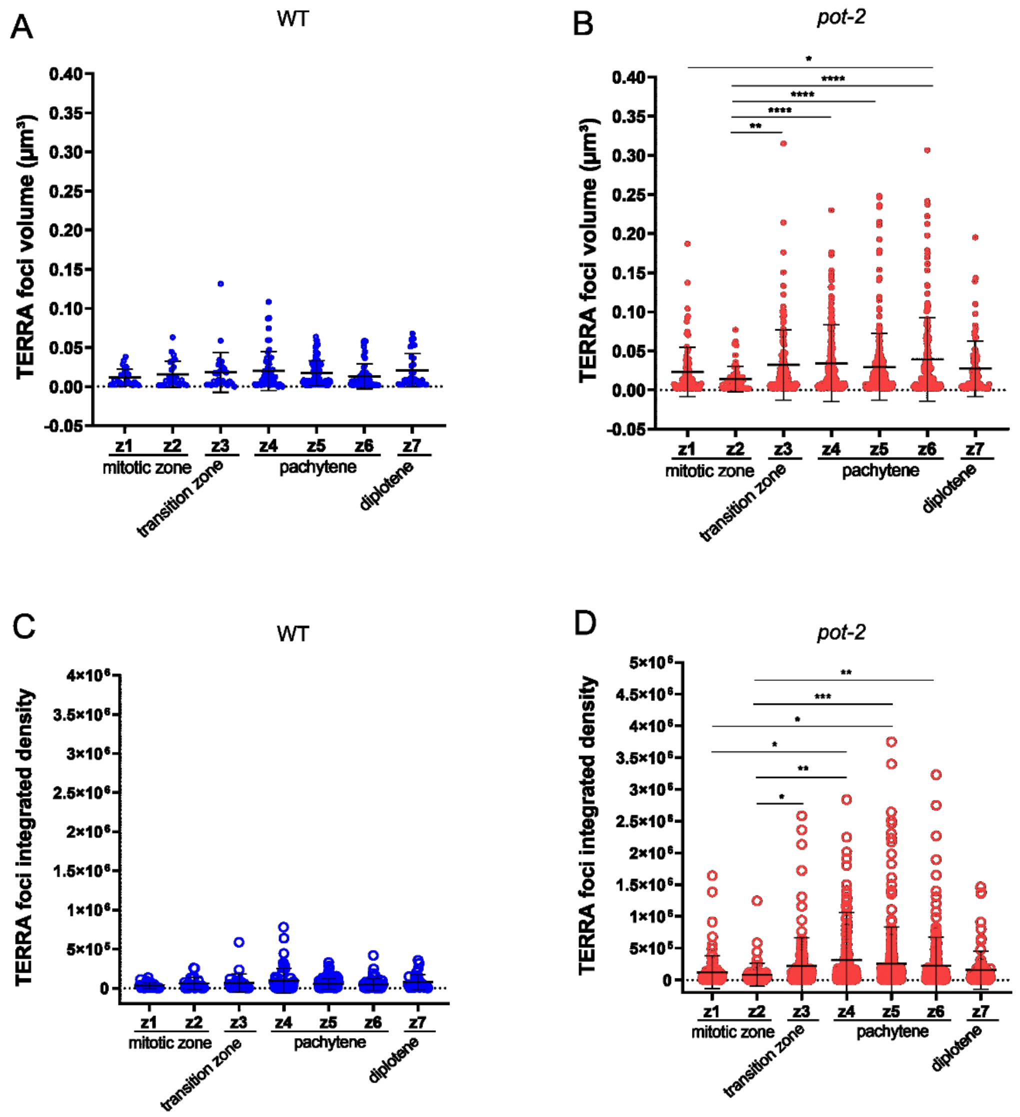

Figure S4. Quantification of the TERRA foci volume and integrated density in germline cells. A-D) Quantification of the volume (A-B) and integrated density (C-D) of TERRA foci detected by RNA FISH in the indicated meiotic zones of the germline from wild type (A,C) and *pot-2* (B,D) organisms. Data shown represents mean ± SD from two independent experiments. At least 240 nuclei were assessed in each experiment for each strain. Statistical analyses were performed using One-way Brown-Forsythe ANOVA test and the Game-Howell's multiple comparison. Column factor (difference between zones 1-7) p-values= ns: not significant (Figures S3A and S3C), ***: <0.001 (Figures S3B and S3D). Multiple comparison p-value: *: <0.05, **: <0.01, ***: <0.001, ****: <0.0001.

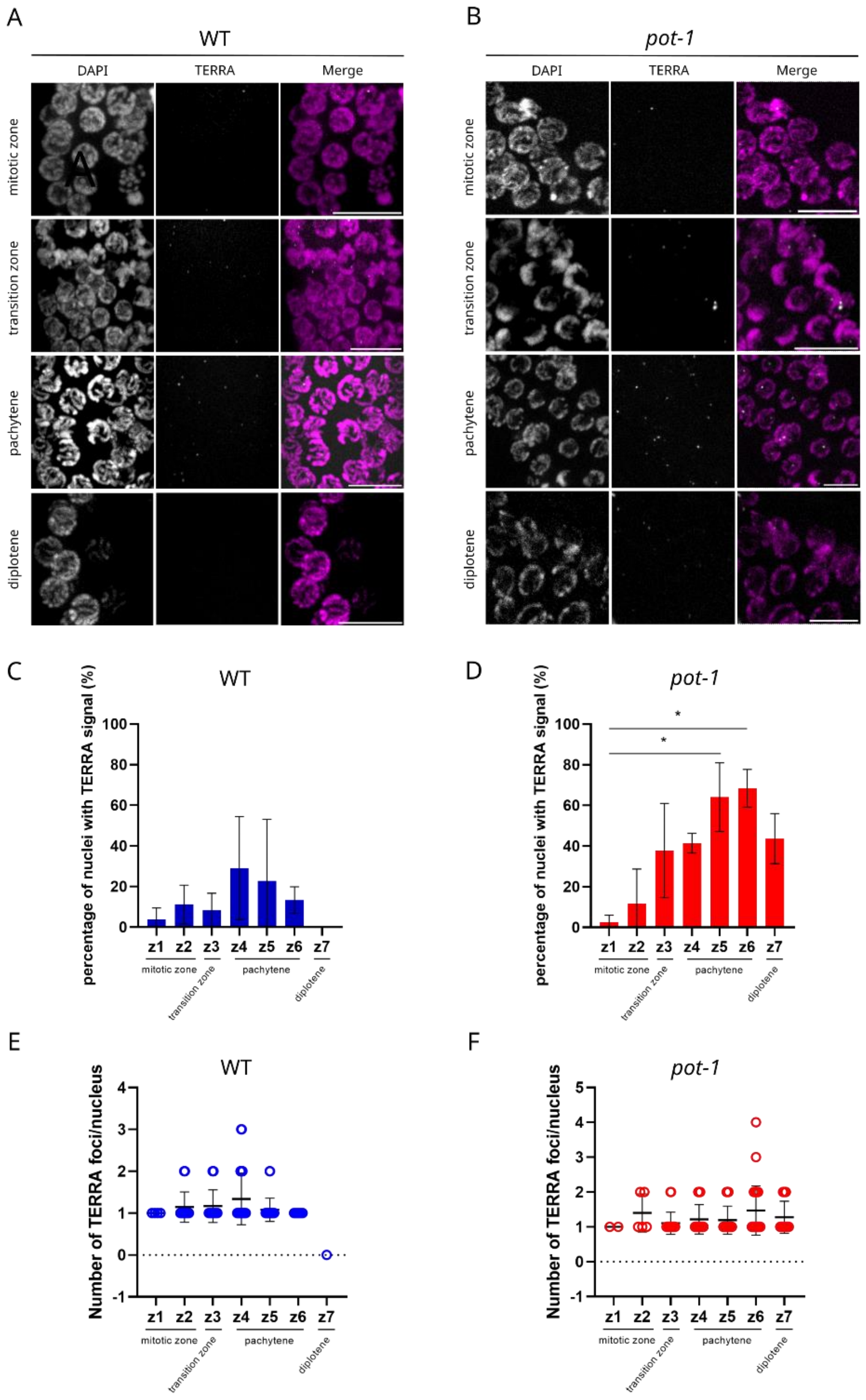

Figure S5. **Increased detection of TERRA foci during pachytene in a *pot-1* mutant strain. A-B)** Detection of TERRA by RNA FISH in the indicated meiotic zones of the germline of WT (A) and *pot-1* (B) organisms. Nuclei

were stained by DAPI. Scale bar: 10 µm. **C-D)** Quantification of the number of nuclei positive to TERRA signal in the different meiotic zones of WT (C) and *pot-1* (D) organisms. Data are shown as percentage of TERRA-positive nuclei and represent mean ± SD from two independent experiments. Four and three gonads were assessed for the WT and *pot-1* mutant, respectively, corresponding to a total of 725 (WT) and 368 (*pot-1*) nuclei analyzed. Statistical tests were performed using the Two-way ANOVA with Tukey's multiple comparison test. P-value: *= ≤ 0.05. **E-F)** Quantification of the number of TERRA foci per nucleus displaying TERRA signal detected by RNA FISH in the indicated meiotic zones of the germline from WT (E) and *pot-1* (F) organisms. The numbers of gonads and nuclei analyzed are as in C-D. Imaging was performed using a spinning disc microscope. For this reason, the absolute values of the quantification analyses shown in this figure are not comparable to the values of the analyses displayed in the main Figure 2 for which a DeltaVision microscope was used for the imaging.

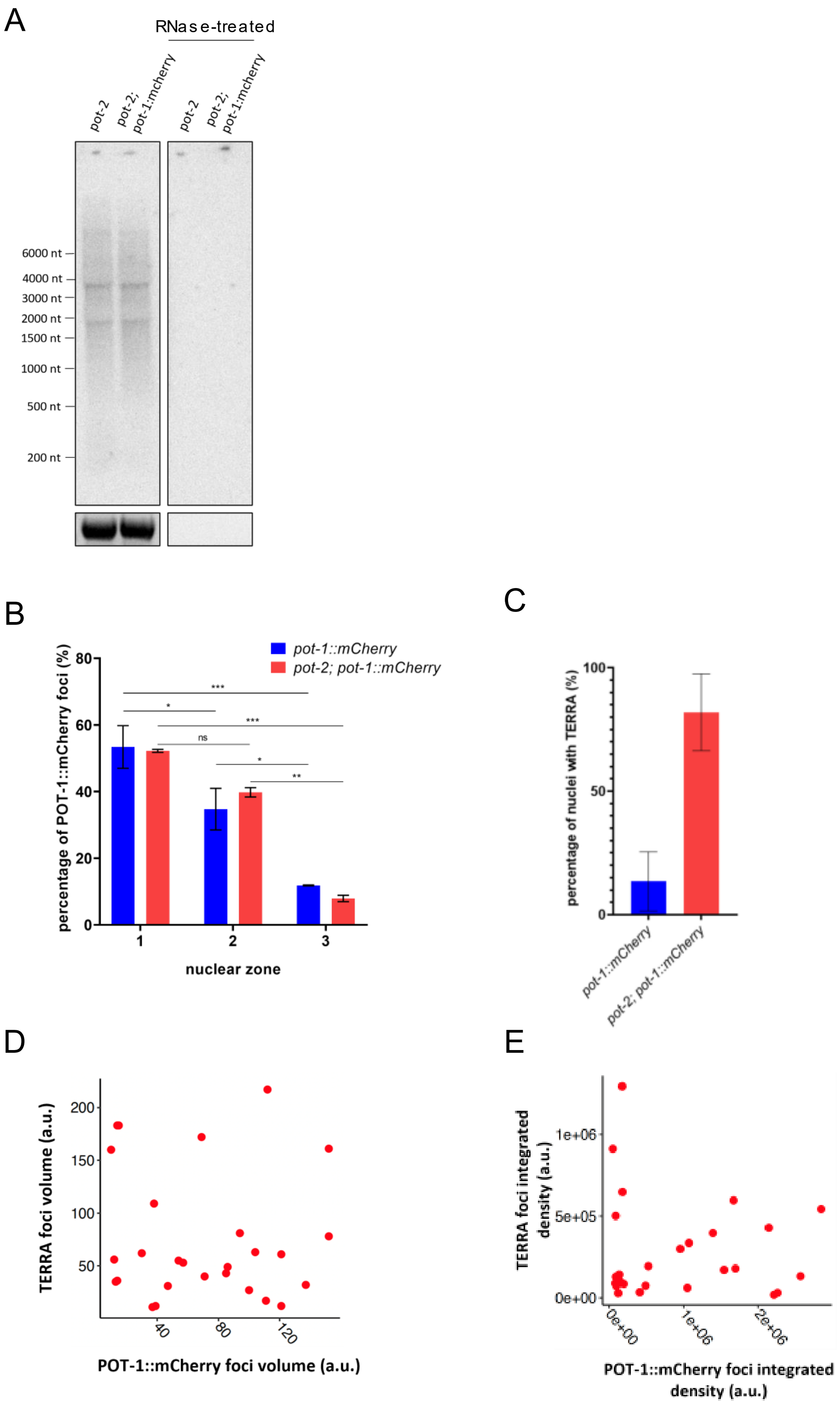

Figure S6. Detection of TERRA and telomeres in *pot-1::mCherry* strains. A) Northern blot analysis of TERRA from *pot-2* and *pot-2; pot-1::mCherry* strains. Total RNA was resolved on agarose gel and membrane hybridization was performed using a radioactively labelled C-rich telomeric probe. Bottom image shows 18S rRNA band upon

gel run. The experiment was repeated two times obtaining similar results. **B)** Quantification of POT-1::mCherry foci detected by anti-mCherry IF in each of the three nuclear zones in mid-pachytene germ cells of *pot-1:mCherry* and *pot-2; pot-1:mCherry* organisms. Data are shown as percentage of foci and represent mean ± SD from two independent experiments. At least 8 nuclei were assessed for each strain in each experiment. Two-way ANOVA with Tukey's multiple comparison test was used for statistical analyses. Column factor (difference between zones 1, 2 and 3) p-value= ****: ≤0.0001; Multiple comparison tests p-value= *: ≤0.05, **: ≤0.01, ***: ≤0.001. **C)** Quantification of the number of germline nuclei positive to TERRA RNA FISH signal in RNA FISH/IF experiments performed in *pot-1::mCherry* and *pot-2*; *pot-1::mCherry* strains. Data are shown as percentage of TERRA-positive nuclei and represent mean ± SD from three independent experiments. At least 8 nuclei were assessed in each experiment. The RNA FISH/IF technique results in attenuation of the TERRA RNA FISH signal due to the IF protocol, with consequent lower number of TERRA foci detected, compared to the RNA FISH technique. **D-E)** Correlation analyses of volumes (D) and integrated densities (E) of TERRA and POT-1::mCherry colocalizing foci in the *pot-2; pot-1::mCherry* strain*.* A total number of 82 TERRA foci detected in mid-pachytene germ cells were analyzed from 5 different gonads in two independent experiments. No significant correlation was detected for either of the two parameters analyzed using Pearson's product-moment correlation. In D p-value= 0.4466, correlation coefficient: -0,1528, R^2: 0,0234; in E p-value= 0.7313, correlation coefficient: -0,0691, R^2: 0,0048.

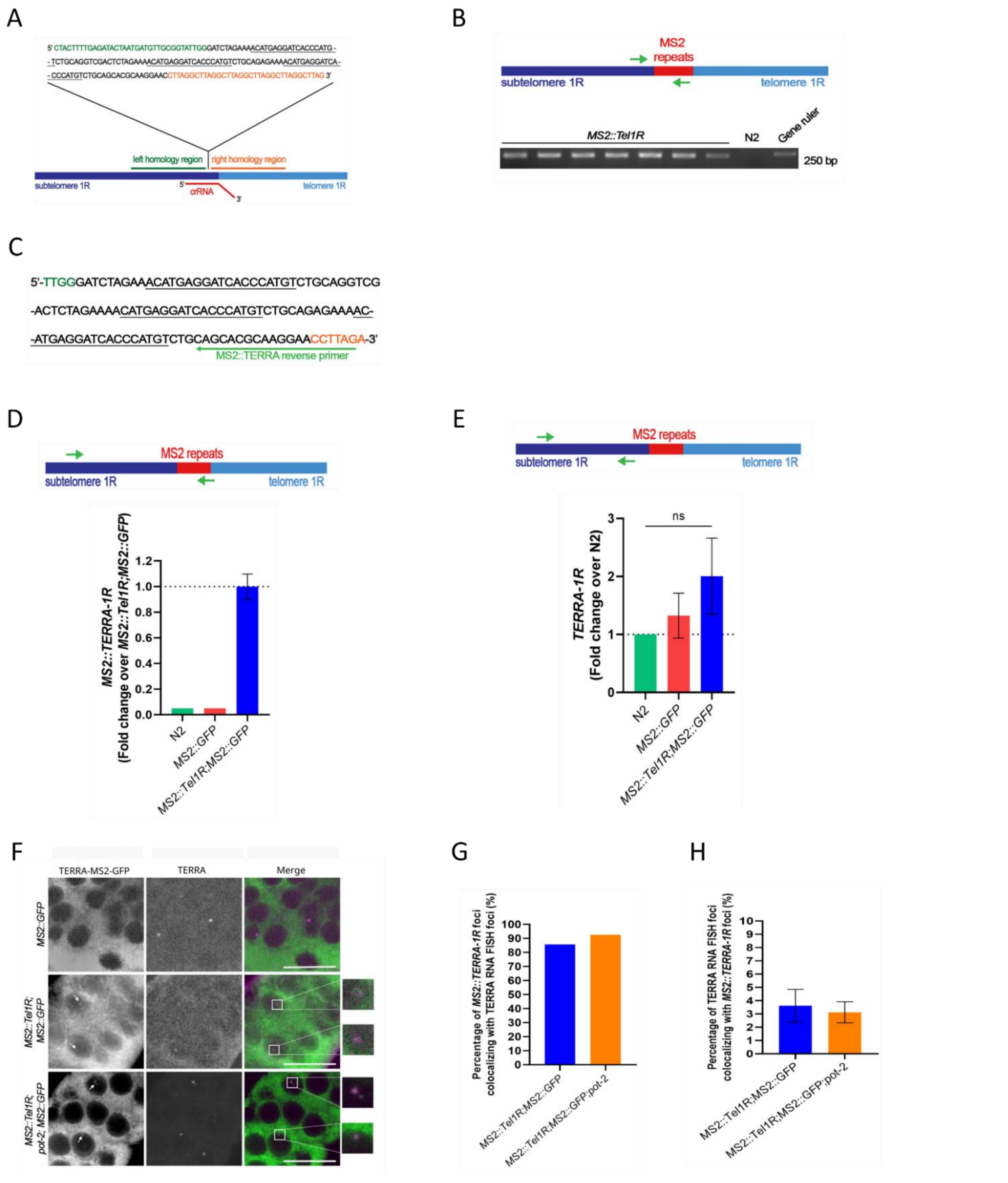

Figure S7. Integration site of the MS2 sequences at subtelomere 1R and verification of MS2-TERRA expression. A) Schematic depiction of the subtelomere 1R showing the sequence of the repair template containing MS2 stem loop sequences, integrated by the mean of the CRISPR/Cas9 system. The repair template contains three MS2 sequences (underlined) flanked by a 5' homology arm, or left homology region (in green), mapping on the subtelomere 1R region and a 3' homology arm, or right homology region (in orange), consisting

of a sequence spanning the last nucleotides of the subtelomere 1R, adjacent to the telomeric repeat tract, and telomeric sequences. **B)** Schematic depiction of the subtelomere 1R containing the MS2 sequences indicating the position of the primers used to genotype the *MS2:Tel1R* strains (MS2::TERRA forward and reverse primers, shown as green arrows). An electrophoretic run of the amplicons obtained from the PCR performed on genomic DNA from *MS2::Tel1R* and wild type strains using the primer pair MS2::TERRA forward and reverse is shown below. The sequences of the primers used in this study for genotyping are indicated in Table S2. **C)** Sequencing result of the amplicon obtained from the PCR performed on the *MS2::Tel1R* strain using the primer pair MS2::TERRA forward and reverse. Sequencing was performed using the MS2::TERRA forward primer. A portion of the left homology arm (sequence in green) and the right homology arm (sequence in orange) are visible at the 5' and 3' of the sequence, respectively. **D)** RT-qPCR analyses of MS2::TERRA-1R in wild type (N2), *MS2::GFP* and *MS2::Tel1R; MS2::GFP* strains. MS2::TERRA-1R transcripts were detected in the *MS2::Tel1R; MS2::GFP* strain only. Data represents mean ± SD from three independent experiments and are shown as $2^{\wedge\text{-Dct}}$ values using *Arp6* as reference gene. A schematic depiction of the subtelomere 1R containing MS2 repeats and the localization of MS2::TERRA-1R qPCR primer pairs (green arrows) is shown on top. **E)** RT-qPCR analyses of TERRA transcripts expressed from subtelomere 1R (TERRA-1R) in wild type (N2), *MS2::GFP* and *MS2::Tel1R; MS2::GFP* strains. TERRA-1R is detected at similar levels in the three strains. Data represents mean ± SD from three independent experiments and are shown as fold change of $2^{\wedge\text{-Dct}}$ values over N2. *Arp6* was used as internal reference gene. A schematic depiction of the subtelomere 1R containing MS2 repeats and the localization of TERRA-1R qPCR primer pairs (green arrows) is shown on top. TERRA-1R primer pairs anneal to the subtelomeric sequence of TERRA within subtelomere 1R regardless of the presence of the MS2 sequences. *Brown-Forsythe* ANOVA test and the *Games Howell's* multiple comparison test were performed to calculate statistics. *ns*= not significant. **F)** RNA FISH/IF experiments to detect total TERRA (TERRA) and MS2::TERRA-1R MS2::GFP (TERRA-MS2-GFP) in the indicated strains. Detection of TERRA-MS2-GFP foci was achieved by IF using anti-GFP antibody; total TERRA was detected by RNA FISH using a C-rich telomere-specific fluorescent probe. Insets show colocalization events between TERRA-MS2-GFP foci and TERRA signal. TERRA-MS2-GFP foci were not detected in the MS2::GFP control strain. Scale bar: 10µm. **G)** Quantification of the number of TERRA-MS2-GFP foci detected by anti-GFP IF colocalizing with TERRA RNA FISH signal. Data are shown as percentage and refer to the analyses of two (*MS2::Tel1R; MS2::GFP* strain) and seven gonads *(MS2::Tel1R; pot-2; MS2::GFP* strain) for a total of 162 and 771 TERRA RNA FISH foci analyzed in each strain, respectively. **H)** Quantification of the number of TERRA foci detected by RNA FISH colocalizing with TERRA-MS2-GFP foci as detected by anti-GFP IF. Data are shown as percentage and represent mean ± SD from the analyses of two (*MS2::Tel1R; MS2::GFP* strain) and seven gonads *(MS2::Tel1R; pot-2; MS2::GFP* strain) for a total of 162 and 771 TERRA RNA FISH foci analyzed in each strain, respectively.

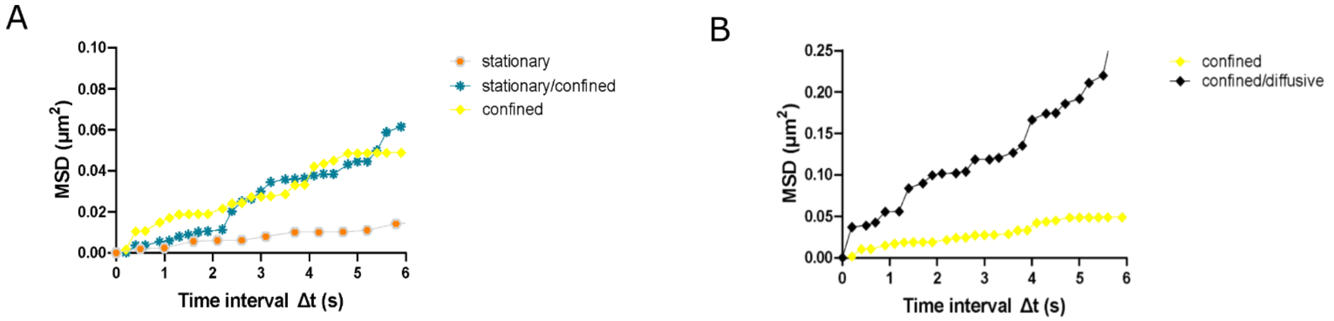

Figure S8. Examples of TERRA particles transiting between different diffusion states. A) Mean-square displacement (MSD) versus time of a tracked TERRA-MS2-GFP particle transiting from stationary to confined motions, as well as of a stationary and a confined particle. The tracking was performed in live *MS2::Tel1R; pot-2; MS2::GFP* organisms using a spinning disc microscope. **B)** Mean-square displacement (MSD) versus time of a tracked TERRA-MS2-GFP particle transiting from confined to diffusive motions and a particle remaining in confined motion throughout the time of acquisition. The tracking was performed in live *MS2::Tel1R; pot-2; MS2::GFP* organisms using a spinning disc confocal microscope. Graphs related to Figure 4C.

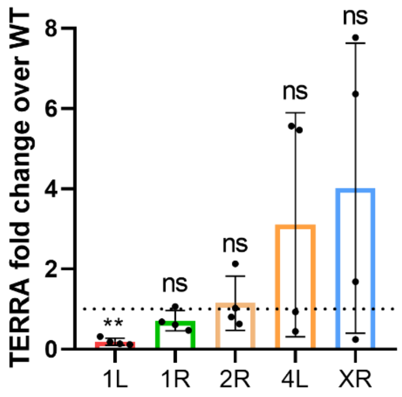

Figure S9. Quantification of TERRA expression from five telomeres in *trt-1; pot-2* mutant versus wild type. RT-qPCR analyses of TERRA from the indicated telomeres in *trt-1; pot-2* (F16). Data are shown as fold change over the wild type strain and represent mean and SD from four independent experiments. Two-way ANOVA test with Tukey's multiple comparison was used to assess statistical analyses. Multiple comparison tests showed significant difference for telomere 1L TERRA expression between *trt-1; pot-2* double mutant and the wild type. p-value= **: ≤0.01. ns= not significant

**Name of the strain**	**Genetic**	**Source**
N2	Wild type, Bristol	Acquired from CGC
pot-1	Allele*: tm1620 III*	Acquired from CGC
pot-2	Allele*: tm1400 II*	Acquired from CGC
trt-1	Allele*: ok410 I*	Acquired from CGC
pot-1::mCherry	Allele**:** *ypSi2 [Pdaz-1::pot-1::mCherry::tbb-2 3'UTR+Cbr-unc-119(+)] II*	Acquired from CGC
JK5896	Alleles*: qSi369 II;* unc-119(ed3) *III; qSi370 V qSi369 [sygl-1p::24xMS2 loops::3xflag::sygl-1::sygl1 3'UTR]. qSi370 [mex-5p:: MS2 Coat Protein::linker::sfGFP::tbb-2 3' UTR::gpd-2 intergenic sequence::H2B::mCherry::unc-54 3' UTR].*	Acquired from CGC
MS2::GFP	Allele**:** *qSi370 [mex-5p:: MS2 Coat Protein::linker::sfGFP::tbb-2 3' UTR::gpd-2 intergenic sequence::H2B::mCherry::unc-54 3' UTR] V*	This study
MS2::Tel1R	Allele*: jf195 [3xMS2 loops::terra] I*	This study
pot-2; pot-1::mCherry	Alleles*: tm1400 II; ypSi2 [Pdaz-1::pot-1::mCherry::tbb-2 3'UTR+Cbr-unc-119(+)] II*	This study
trt-1; pot-2; pot-1::mCherry	Alleles*: ok410 I; tm1400 II; ypSi2 [Pdaz-1::pot-1::mCherry::tbb-2 3'UTR+Cbr-unc-119(+)] II*	This study
trt-1; pot-2	*Alleles: ok410 I; tm1400 II*	This study
trt-1; pot-1::mCherry	Alleles*: ok410 I; ypSi2 [Pdaz-1::pot-1::mCherry::tbb-2 3'UTR+Cbr-unc-119(+)] II*	This study
MS2::Tel1R; MS2::GFP	Alleles*: jf195 [3xMS2 loops::terra] I; qSi370 [mex-5p:: MS2 Coat Protein::linker::sfGFP::tbb-2 3' UTR::gpd-2 intergenic sequence::H2B::mCherry::unc-54 3' UTR] V*	This study
MS2::Tel1R; pot-2; MS2::GFP	Alleles*: jf195 [3xMS2 loops::terra] I; tm1400 II; qSi370 [mex-5p:: MS2 Coat Protein::linker::sfGFP::tbb-2 3' UTR::gpd-2 intergenic sequence::H2B::mCherry::unc-54 3' UTR] V*	This study
MS2::Tel1R; trt-1; MS2::GFP	Alleles*: jf195 [3xMS2 loops::terra] I; qSi370 [mex-5p:: MS2 Coat; ok410 I Protein::linker::sfGFP::tbb-2 3' UTR::gpd-2 intergenic sequence::H2B::mCherry::unc-54 3' UTR] V*	This study
MS2::Tel1R; trt-1; pot-2; MS2::GFP	Alleles*: jf195 [3xMS2 loops::terra] I; qSi370 [mex-5p:: MS2 Coat; ok410 I; tm1400 II Protein::linker::sfGFP::tbb-2 3' UTR::gpd-2 intergenic sequence::H2B::mCherry::unc-54 3' UTR] V*	This study
MS2::Tel1R; trt-1	Alleles*: jf195 [3xMS2 loops::terra] I; ok410 I*	This study
pot-1; pot-2	Alleles*: tm1620 III; tm1400 II*	This study

Table S1. List of strains used in this study.

Allele	Primer name	Sense	Primer sequence
jf195 [3xMS2 loops::terra] I	MS2::TERRA forward	forward	5'-TCGGTTGTTGCATCTCTACTTT-3'
	MS2::TERRA reverse	reverse	5'-CTAAGGTTCCTTGCGTGCTG-3'
ypSi2[Pdaz-1::pot-1::mCherry::tbb-2 3'UTR+Cbr-unc-119(+)]II	Mos_1	wildtype reverse	5'-CGTTCATACGGCCGAAATTT-3'
	Mos_2	common forward	5'-CAAGACACCCGGGTTTGTCT-3'
	Mos_3	mutant reverse	5'-CAATTCATCCCGGTTTCTGT-3'
pot-2(tm1400)	pot-2_F	forward	5'-AAGTTCATTCGGTTGTCGAA-3'
	pot-2_R	reverse	5'-CAATTGTCCAACTCCATATCCA-3'
pot-1 (tm1620)	pot-1_F	common forward	5'-TGCAATACACTTACCAGCACA -3'
	pot-1_R1	wildtype reverse	5'-CTGACCCTTTCACTGTCTTCA-3'
	pot-1_R2	mutant reverse	5'-TGAACGGAACACTACCAACC-3'
trt-1 (ok410)	trt-1_A	common forward	5'-ATGGTTGAAATCGAGTGAGCT-3'
	trt-1_B	wildtype reverse	5'-TGTCGTAAAGCCATCAGAGC-3'
	trt-1_C	mutant reverse	5'-TCACTCCACACCATTGAAGG-3'
qSi370 [mex-5p:: MS2 Coat Protein::linker::sfGFP::tbb-2 3' UTR::gpd-2 intergenic sequence::H2B::mCherry::unc-54 3' UTR] V	Mos_2	forward	5'-CAAGACACCCGGGTTTGTCT-3'
	sfGFP_R	reverse	5'-TGTGTCCGAGGATGTTTCCG-3'

Table S2. List of primers used in this study for genotyping.

Locus	Sense	Sequence
subtelomere 1L	Forward	5’ – AAGCCTAAAAAATTGAGATAAGAAAACAT – 3’
subtelomere 1L	Reverse	5’ – AGGCAGGCAAAATTAGAGGTAC – 3’
subtelomere 1R	Forward	5’ – AAGCCTAAGACCAATACCGCAAC – 3’
subtelomere 1R	Reverse	5’ – GTTTCGGTTGTTGCATCTCTAC – 3’
subtelomere 2R	Forward	5’- GCTTGACCATAAACTCCCCT – 3’
subtelomere 2R	Reverse	5’- GAAAAGCTCCCGGGTCTTAC – 3’
subtelomere 3R	Forward	5’ – ACTGAGCTTTTCCATCCTCGT – 3’
subtelomere 3R	Reverse	5’ – AACAAACGCGGTGCGGAG – 3’
subtelomere 4L	Forward	5’ – AAGCCTAAGAAGAGACCAAACC– 3’
subtelomere 4L	Reverse	5’ – GCGAAAGATGAATGTTCAAAGC – 3’
subtelomere XR	Forward	5’ – GCCTGAAAATTCTCATTATTCGATAG – 3’
subtelomere XR	Reverse	5’ – TTGATGTGACCAATTGTACTTTTCC – 3’
MS2::TERRA	Forward	5’ – TCGGTTGTTGCATCTCTACTTT – 3’
MS2::TERRA	Reverse	5’ – CTAAGGTTCCTTGCGTGCTG – 3’
arp-6	Forward	5’ – AACCATCTACGACGAATCGCT – 3’
arp-6	Reverse	5’ – CATGCATTTCAGCTTTTCAGTGATG – 3’
tba-1	Forward	5’ – TCAACACTGCCATCGCCGCC – 3’
tba-1	Reverse	5’ – TCCAAGCGAGACCAGGCTTCA – 3’

Table S3. List of primers used in this study for qPCR.

**Primer pair**	**slope**	**R^2**	**efficiency**
1L	-3,329	0,9960	99,7%
1R	-3,375	0,9972	97,8%
2R	-3,388	0,9558	97,3%
3L	-3,270	0,9922	102,2%
4R	-3,323	0,9949	99,9%
XR	-3,542	0,9956	91,5%
arp-6	-3,263	0,9919	102,5%
tba-1	-3,251	0,9753	103,0%

Table S4. Amplification efficiencies of TERRA primer pairs analyzed from qPCR assays performed on dilutions of genomic DNA (200ng, 20ng, 2ng, 0.2ng, 0.02ng and 0.002ng) extracted from N2 organisms. The log10 of genomic DNA concentrations were plotted as function of the Ct values. Primer efficiencies were calculated as described in the materials and methods section.

**Antibody**	**Source**	**Identifier**	**Dilution**
Rabbit anti-mCherry	Kindly provided by Alex Dammermann, Max Perutz Labs, Vienna	-	1:400
Rabbit anti-mCherry	Invitrogen	#PA5-34974	1:500
Mouse anti-GFP	ThermoFisher Sceintific	#A-11122	1:500
Goat anti-Rabbit IgG (H+L) Cross-Adsorbed Secondary Antibody, Alexa Fluor 568	Invitrogen	#A-11036	1:500
Goat anti-Rabbit IgG (H+L) Cross-Adsorbed Secondary Antibody, Alexa Fluor 555	Invitrogen	#A-21428	1:1500
Goat anti-Mouse IgG (H+L) Highly Cross-Adsorbed Secondary Antibody, Alexa Fluor Plus 488	Invitrogen	#A32723	1:1500

Table S5. List of antibodies used in this study. Source and dilution used in immunofluorescence experiments are shown.
